# Genome-wide mapping reveals single-origin chromosome replication in *Leishmania*, a eukaryotic microbe

**DOI:** 10.1186/s13059-015-0788-9

**Published:** 2015-10-19

**Authors:** Catarina A. Marques, Nicholas J. Dickens, Daniel Paape, Samantha J. Campbell, Richard McCulloch

**Affiliations:** The Wellcome Trust Centre for Molecular Parasitology, Institute of Infection, Immunity and Inflammation, University of Glasgow, Sir Graeme Davis Building, 120 University Place, Glasgow, G12 8TA UK

## Abstract

**Background:**

DNA replication initiates on defined genome sites, termed origins. Origin usage appears to follow common rules in the eukaryotic organisms examined to date: all chromosomes are replicated from multiple origins, which display variations in firing efficiency and are selected from a larger pool of potential origins. To ask if these features of DNA replication are true of all eukaryotes, we describe genome-wide origin mapping in the parasite *Leishmania*.

**Results:**

Origin mapping in *Leishmania* suggests a striking divergence in origin usage relative to characterized eukaryotes, since each chromosome appears to be replicated from a single origin. By comparing two species of *Leishmania*, we find evidence that such origin singularity is maintained in the face of chromosome fusion or fission events during evolution. Mapping *Leishmania* origins suggests that all origins fire with equal efficiency, and that the genomic sites occupied by origins differ from related non-origins sites. Finally, we provide evidence that origin location in *Leishmania* displays striking conservation with *Trypanosoma brucei*, despite the latter parasite replicating its chromosomes from multiple, variable strength origins.

**Conclusions:**

The demonstration of chromosome replication for a single origin in *Leishmania*, a microbial eukaryote, has implications for the evolution of origin multiplicity and associated controls, and may explain the pervasive aneuploidy that characterizes *Leishmania* chromosome architecture.

**Electronic supplementary material:**

The online version of this article (doi:10.1186/s13059-015-0788-9) contains supplementary material, which is available to authorized users.

## Background

The earliest stage of DNA replication is the designation of defined genome sites, termed origins, where DNA synthesis initiates. Origins are binding sites for replication initiator factors, which mediate recruitment of the replication machinery [1]. Despite the fundamental role of DNA replication in life, origin structure and usage is not conserved across biology [2]. The genomes of most bacteria and many archaea are replicated from single origins, which fire in every replication cycle, display sequence conservation within each kingdom and, at least in bacteria, are largely conserved in genome position [3]. In contrast, in every eukaryote examined to date each linear chromosome is replicated from multiple origins that display variations in frequency and timing of firing. Moreover, identification of a consensus origin sequence amongst the multiple mapped sites has proved impossible in nearly all eukaryotes, with the exception of *Saccharomyces* yeast and its relatives [4].

The kinetoplastida is a well-studied order of eukaryotic microbes that contains a number of notable human and animal parasites, including *Leishmania* and *Trypanosoma*. Nuclear gene expression in kinetoplastids is unusual amongst eukaryotes, since virtually all genes are arranged in a small number (~200) of multigene transcription units, each of which is transcribed from a single promoter. As a result, the number of promoters and transcription termination sites in kinetoplastid genomes is only around 1–2 % of the gene number, with gene expression primarily controlled by post-transcriptional processes. RNA polymerase (RNA Pol) II promoters are poorly understood in kinetoplastids, but are at loci termed strand switch regions (SSRs), which are marked by modified histone enrichment [5, 6] and found where transcription units diverge (divergent SSRs) or are orientated head-to-tail (H-T SSRs). Transcription termination is also poorly understood, though loss of a modified base (J) causes RNA Pol read-through at convergent and H-T SSRs in *Leishmania* [7, 8]. Previously, we mapped origins in the *Trypanosoma brucei* genome using MFAseq (or Sort-seq), which compares DNA read depth across each chromosome in replicating cells relative to non-replicating cells [9, 10]. Allied to localisation of an initiator factor, ORC1/CDC6 [9, 11, 12], *T. brucei* replication appears to fit many of the eukaryotic paradigms [13, 14], despite the unusual genetic landscape: each chromosome is replicated from more than one origin; origins are selected from a larger pool of ORC1/CDC6 binding sites, suggesting redundancy and perhaps dormancy; and origin strength is non-uniform, suggesting a temporal order of firing or variable levels of origin usage within the population. Moreover, though *T. brucei* ORC1/CDC6 binds at potentially all SSRs, we have been unable to identify consensus binding sequences; indeed, other than the centromeres [9, 15], we cannot distinguish between origin-active and non-active ORC1/CDC6 binding sites.

Here, we describe mapping of replication initiation in two *Leishmania* species. Our rationale was that comparing *Trypanosoma* and *Leishmania* could provide insight into origin function and conservation. The two genera diverged ~250 million years ago [16] and have evolved distinct strategies for parasitism, survival and transmission. However, despite this divergence, the parasites’ genomes display considerable synteny, with ~70 % of genes in *Leishmania major* and *T. brucei* found in the same genomic context [17]. Remarkably, such genome synteny is found in the context of pronounced structural and functional differences, since the *L. major* genome is composed of 36 chromosomes (size range ~0.2–2.5 Mb), compared with 11 in *T. brucei* (~1.0–5.0 Mb). In addition, *L. major* chromosomes lack the large, highly variable subtelomeres found in *T. brucei*. Finally, genome stability is variant between the genera: whereas the *T. brucei* chromosomes appear to be stably diploid, aneuploidy is a pervasive feature of all *Leishmania* species, with multiple chromosomes seen in non-diploid configurations in parasite populations and ploidy changes of individual chromosomes detectable between cells in a population [18]. Whether or not aneuploidy in *Leishmania* is mechanistically related to gene copy number variation and gene amplification, in some cases allowing adaptive changes in gene expression, remains unclear [19, 20]. By mapping replication origins in *Leishmania*, we show that there is considerable conservation of location, though not origin sequence, relative to *T. brucei*. However, origin usage in *Leishmania* is strikingly different from *T. brucei* and all other characterised eukaryotes, with only a single detectable origin per chromosome. Such unorthodox eukaryotic origin usage is associated with uniform origin strength and origins being found at specific genomic loci, which provides insight into the evolution of origin multiplicity and associated controls, and has implications for genome maintenance.

## Results

### Single origins of replication in each chromosome of *L. major*

**Fig. 1:**
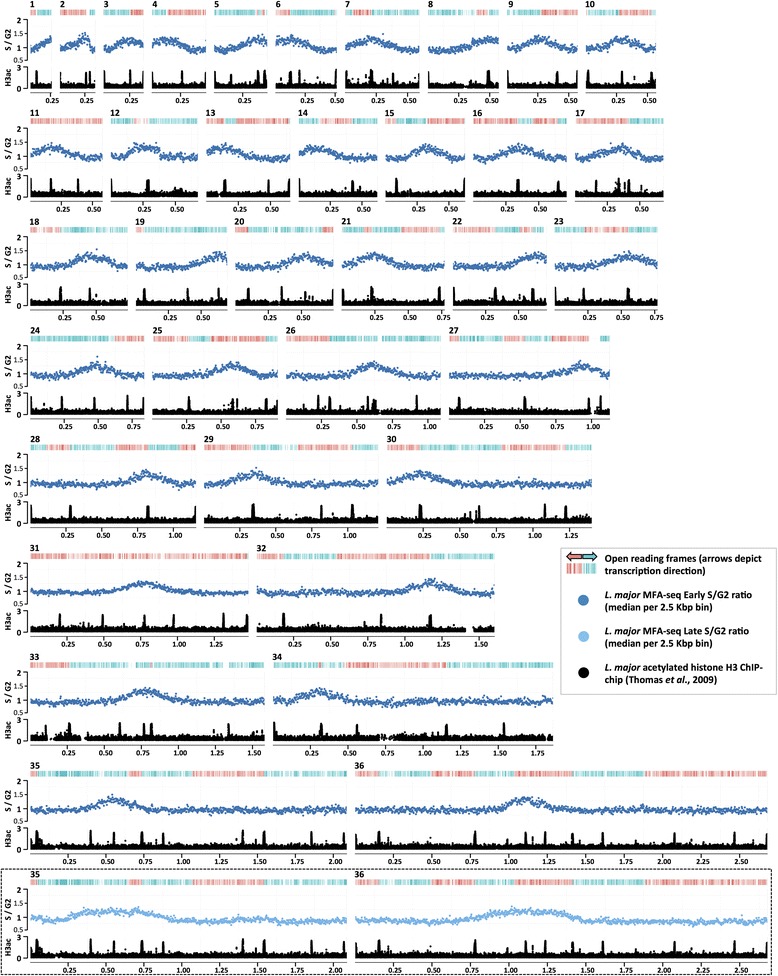
Mapping replication origins in the *L. major* nuclear genome. Graphs show the distribution of replication origins in the 36 chromosomes of *L. major* (numbered 1–36; sizes denoted in intervals of 0.25 Mb), determined by the extent of enrichment of DNA in S phase relative to G2. For each chromosome, the top track displays coding sequences, with genes transcribed and translated from right to left in *red*, and from left to right in *blue*. The graph below shows the ratio of the read depth between early S phase and G2 samples (y-axis), where each *dot* (*dark blue*) represents the median S/G2 ratio (y-axis) in a 2.5 Kbp window across the chromosome (x-axis). Finally, the track below the graph displays localization of acetylated histone H3 (*H3ac*) in each chromosome (data from [6]), identifying positions of transcription start sites (y-axis; values represented as log2). The insert diagram (*boxed*) shows S/G2 read depth ratio (*light blue dots*) for chromosomes 35 and 36, as above, but here comparing late S cells with G2. (Late S/G2 MFAseq for all *L. major* chromosomes is shown in Figure S2 in Additional file 1)

Genome-wide MFAseq of *L. major* promastigote cells is shown in Fig. 1. Peaks represent sequences enriched in early–mid S phase cells relative to G2 cells (Figure S1 in Additional file 1). Strikingly, we detected only a single MFAseq peak per chromosome, suggesting a single origin per molecule. No further MFAseq peaks were seen when mid–late S phase cells were analysed (Fig. 1; Figure S2 in Additional file 1), suggesting there are no late-firing origins; instead, the width of each peak widened (at least for the larger chromosomes) relative to early S, consistent with replication having proceeded further from the single origin. In addition, single MFAseq peaks per chromosome were also seen when S phase DNA was compared with G1, rather than G2 (Figure S3 in Additional file 1). As in *T. brucei* [9], the locations of all origins correspond with SSRs. Transcription initiation loci in *Leishmania* are enriched in acetylated histone H3 (H3Ac) [6], and 30 of the 36 origins co-localised with these sites. Five further origins were at convergent SSRs, while one was at the end of chromosome 1, localising either to the transcription termination site or the telomere. From 171 predicted multigene transcription units in *L. major*, origins were found at 21 % of the boundaries, a very similar proportion as seen in *T. brucei* (26 % of 158) [9]. Whether origins are limited to RNA Pol II boundaries is unclear, since on chromosome 27 the origin localised to a divergent SSR from which an RNA Pol II transcription unit and the RNA Pol I transcribed *rRNA* genes emanate. Some of the sites of transcription initiation or termination are associated with RNA Pol III genes (typically tRNAs), but there was no clear distinction between the presence of this transcription in the origin locations relative to SSRs where no origin activity was mapped (data not shown).

### Origin singularity is conserved in *Leishmania* after chromosome fusion or fission

**Fig. 2:**
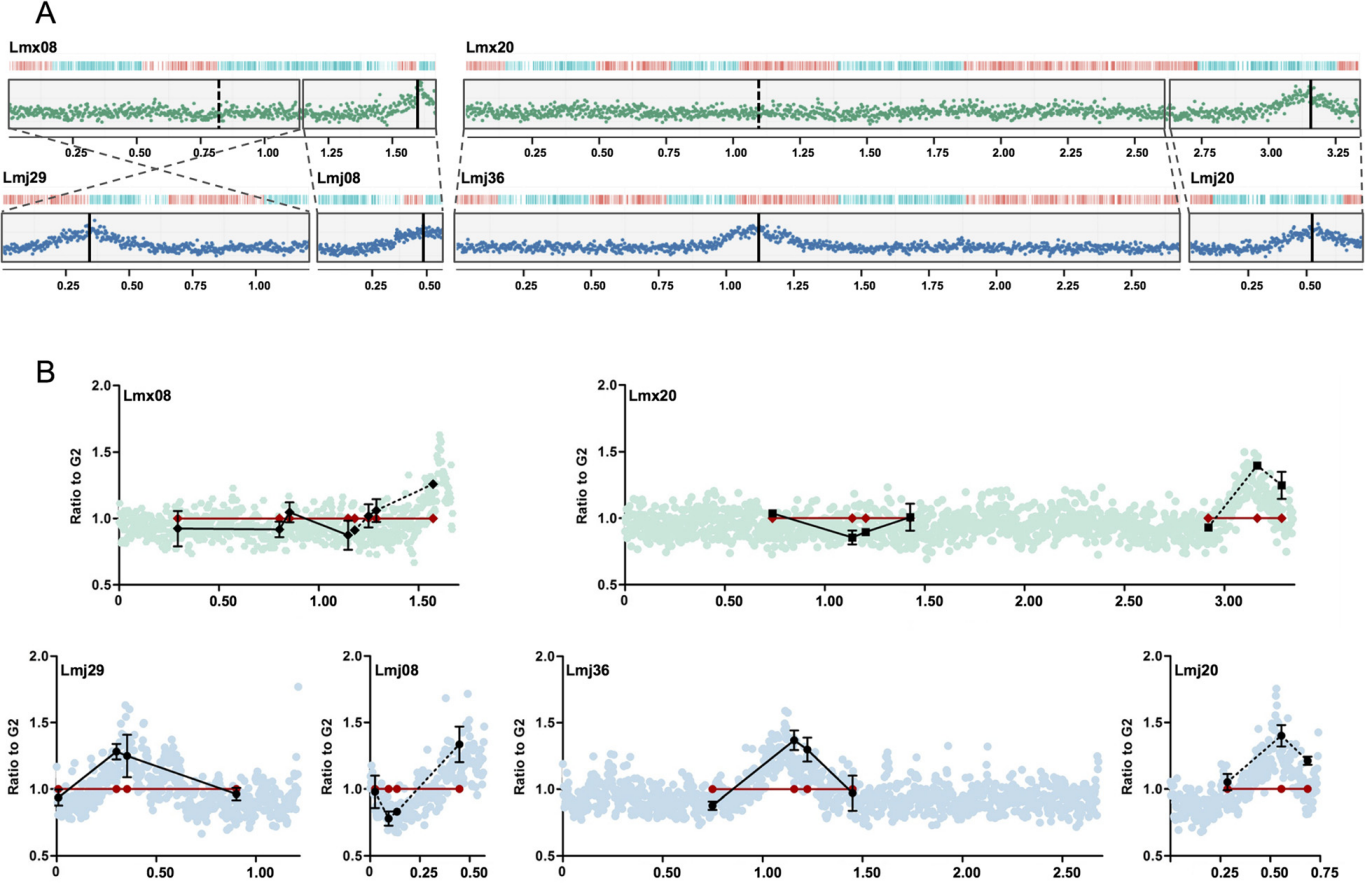
Comparing replication origin usage in syntenic *L. mexicana* and *L. major* chromosomes that have undergone fusion or fission. **a** Graphs show replication origin localisation, evaluated by MFAseq, in *L. mexicana* (*Lmx*) chromosomes 8 and 20, which are syntenic with *L. major* (*Lmj*) chromosomes 29 and 8 and chromosomes 36 and 20, respectively (chromosome sizes are denoted in 0.25 Mb intervals). Blocks of synteny are boxed and their relative orientation indicated; the representation of early S/G2 DNA sequence read depth ratios (*L. mexicana green*, *L. major blue*) and coding sequence organisation are as detailed in Fig. 1 and the approximate location of the origin or syntenic non-origin loci is shown by *solid vertical lines* and *dotted vertical lines*, respectively. (Figures S4 and S5 in Additional file 1 show MFAseq for all *L. mexicana* chromosomes and a genome-wide comparison with *L. major*.) **b** Validation of replication origin activity in the *L. mexicana* and *L. major* chromosomes (shown in (**a**)) by quantitative PCR, which was performed at a number of loci predicted to display origin activity in *L. major* and syntenic with *L. mexicana*. At each locus the relative quantity of S phase (*black*) and G2 phase (*red*) DNA is shown: G2 values at each loci are set at 1, and the S phase samples are shown as a proportion of that value (*vertical lines* indicate standard deviation from at least three experimental repeats); for comparison, the MFAseq data (from (**a**)) is shown in the background, and the right-hand synteny regions are distinguished from the left hand regions using *dotted lines* and *solid lines*, respectively. Positions of the quantitative PCR loci in each chromosome are shown in megabases (x-axes)

Replication of eukaryotic linear chromosomes from a single origin is unprecedented, and so we examined *Leishmania mexicana*, which diverged from *L. major* ~16 million years ago [16]. The genome sizes of the two species are nearly identical (32–33 Mb), but *L. mexicana* contains two fewer chromosomes than *L. major*, due to chromosome fusion or fission: chromosomes 8 and 20 in *L. mexicana* are syntenic with *L. major* chromosomes 8 and 29 and chromosomes 20 and 36, respectively [19]. MFAseq (for both early and late S cells) revealed only a single detectable origin in each *L. mexicana* chromosome (Figures S4 and S5 in Additional file 1), including 8 and 20 (Fig. 2a), both in early and late S phase cells. Origin location was syntenic for 34 of the 36 *L. major* chromosomes (Figure S4 in Additional file 1), suggesting that the same SSRs are used. In contrast, the single origins detected in *L. major* chromosomes 29 and 36 (locations ~0.4 and ~1.1 Mb) did not display MFAseq peaks at the equivalent SSRs on *L. mexicana* chromosomes 8 and 20 (locations ~0.7 Mb and ~1.1 Mb), despite clear synteny in the surrounding genes (Fig. 2a). These data, validated by quantitative real-time PCR (Fig. 2b), suggest that putative chromosome origin singularity is maintained in *Leishmania* even in the face of changing chromosome architecture.

### Substantial conservation of origin location between *L. major* and *T. brucei*

**Fig. 3:**
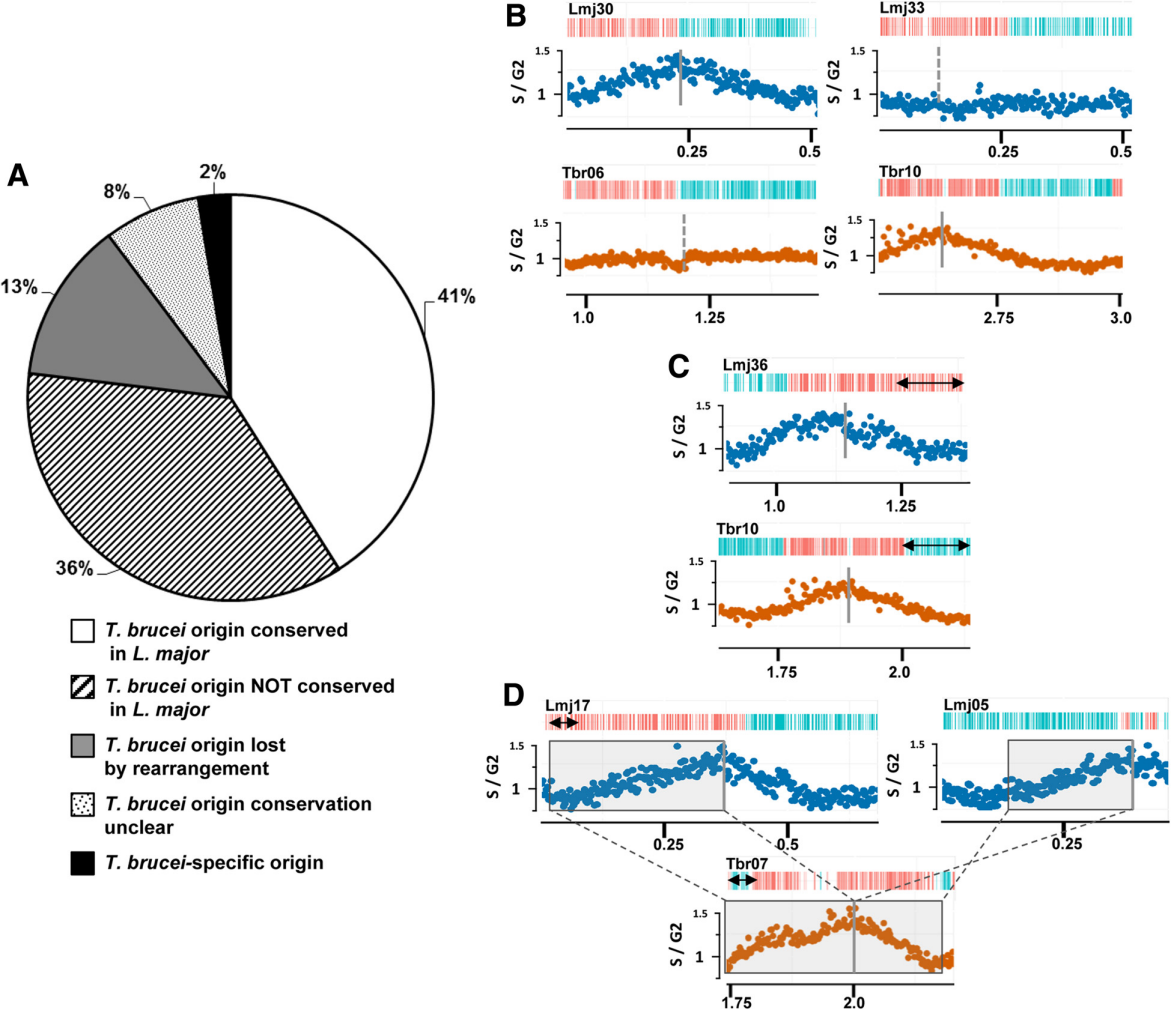
Conservation of DNA replication origins between *L. major* and *T. brucei*. **a** A pie chart showing the proportion of origins mapped in the genome of *T. brucei* whose locations are either conserved or not in the genome of *L. major*, based on whole-genome synteny block comparisons (Figure S6 in Additional file 1). *White* indicates mapped origins in both *T. brucei* and *L. major* within regions of conserved gene synteny; *stripes* indicate mapped *T. brucei* origins within regions of gene synteny in *L .major*, but where no origin activity is mapped in the latter; *grey* indicates *T. brucei* origins at sites of rearrangement relative to *L. major*, where synteny is lost; *dots* depict *T. brucei* origins in regions of synteny with *L. major*, but where local rearrangements mean origin conservation is unclear; *black* represents the single *T. brucei*-specific origin, found in the subtelomere of chromosome 6, which shows no synteny with *L. major*. **b** Synteny conservation between *L. major* (*Lmj*) chromosomes 30 and 33, and *T. brucei* (*Tbr*) chromosomes 6 and 10, respectively, where origin activity is seen in only one of the parasite chromosomes; S/G2 DNA sequence depth ratios (*L. major blue*, *T. brucei orange*) and coding sequence organisation are as detailed in Fig. 1; locations of the regions within the chromosomes are shown in megabases, and the approximate location of the origin or syntenic non-origin loci shown by *solid vertical lines* and *dotted vertical lines*, respectively; *double-headed arrows* denote local rearrangements. **c** An example of a syntenic region between *L. major* chromosome 36 and *T. brucei* chromosome 10 where replication origin activity is conserved. **d** An example of complex origin conservation: a region of *T. brucei* chromosome 7 is shown in which a single origin appears to be conserved as two origins in *L. major* (one origin in two chromosomes: 17 and 5). Synteny blocks are boxed and their relative orientation indicated

Conservation of most origin locations between *L. mexicana* and *L. major* compares well with comparisons of *Saccharomyces* species that diverged at a similar time [21]. However, how conserved are origins between *Leishmania* and *T. brucei*, which are separated by perhaps 20-fold greater evolutionary distance and have highly dissimilar genome architecture? To address this, we built upon previous synteny block analysis [17] and compared origin location in the *T. brucei* and *L. major* genomes (Figure S6 in Additional file 1; summarised in Fig. 3a). Approximately 40 % of origins were conserved in location (i.e., were mapped to SSRs in *T. brucei* and *L. major* located within regions of gene synteny; example in Fig. 3c), while ~35 % of origin-containing SSR loci in *T. brucei* were syntenically conserved in *L. major* but did not display origin activity in the latter parasite (an example is shown in Fig. 3b, as well as an example of a syntenic origin-active SSR in *L. major* that does not display origin activity in *T. brucei*). Only one origin, in the subtelomere of *T. brucei* chromosome 6 (Figure S6 in Additional file 1), appeared to have evolved specifically in that genome. Frequently, origins appeared to be at sites of rearrangement, since 14 origins in *T. brucei* (33 %) were at locations of chromosome fusion or fission relative to *L. major*. In five cases, rearrangements resulted in loss of origin activity in *L. major*, but in the others origin activity was retained, including two instances where a putative single *T. brucei* origin was conserved on two *L. major* chromosomes (example in Fig. 3d). These data contrast with analysis of origin conservation between the budding yeasts *Saccharomyces cerevisiae* and *Lachanacea walti* (~150 million years diverged), where origin sequences are conserved but genomic location is poorly conserved, with location retained for only 12–21 % of origins [4]. The greater conservation of origin location in kinetoplastid genomes may be because multigenic transcription imposes a greater constraint on genome rearrangement, and hence on origin movement.

### Origin usage differs between *Leishmania* and *T. brucei*

**Fig. 4:**
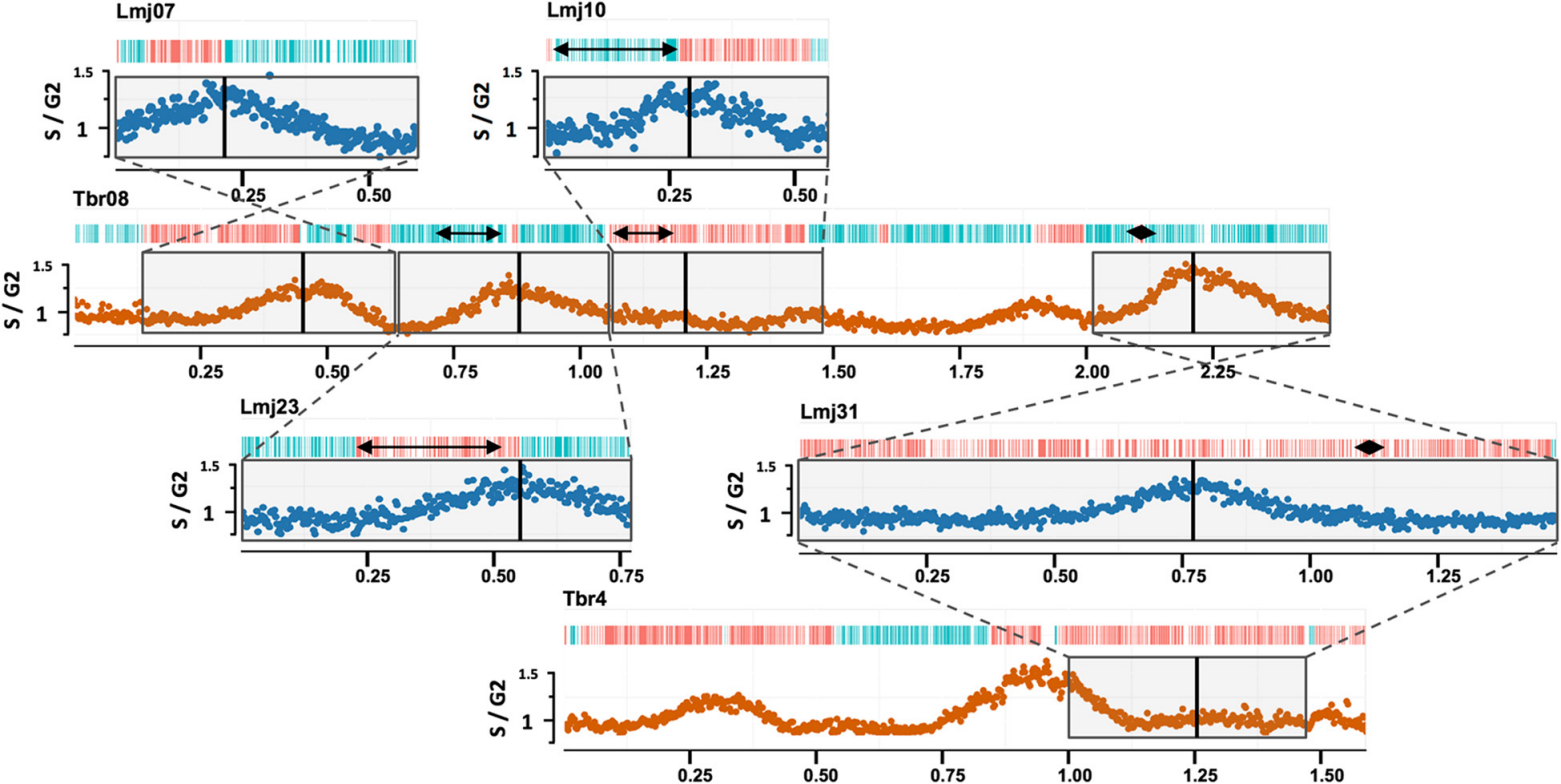
Origin usage is not equivalent in *Leishmania* and *T. brucei*. Synteny conservation is shown between *T. brucei* (*Tbr*) chromosome 8 and *L. major* (*Lmj*) chromosomes 7, 10 and 23, and between *L. major* (*Lmj*) chromosome 31 and *T. brucei* (*Tbr*) chromosomes 4 and 8, comparing the relative strength of the replication origins found within these chromosomes. S/G2 DNA sequence depth ratios and coding sequence organisation are as detailed in Fig. 1. Synteny blocks are boxed and their relative orientation indicated; the approximate location of the origins is shown by *vertical lines. Double-headed arrows denote local rearrangements*

Though the above data suggest considerable conservation of origin location, origin usage is profoundly different between *Leishmania* and *T. brucei*. Scrutiny of the MFAseq peaks revealed considerable uniformity in height and width in both *Leishmania* species (Fig. 1; Figures S2–S5 in Additional file 1). In the majority of *Leishmania* chromosomes the MFAseq peak amplitude (Table S1 in Additional file 1) was close to 0.7 (*L. major* average 0.71, range 0.58–0.82; excluding chromosomes 1, 2, 3, 4, 6, 8, 14, 18 and 20, where MFAseq indicates an origin close to the telomere, limiting analysis of the peak). In contrast, *T. brucei* MFAseq peak amplitudes range from 0.1–0.8 (examples in Fig. 4; Figure S7 in Additional file 1) across and within chromosomes [9], which is very comparable with peak variation seen in *S. cerevisiae* MFAseq analysis [10, 22]. For 24 of the 36 *L. major* chromosomes, where origins were found centrally, the width of the early S MFAseq peaks was very constant (~0.4 Mb; Fig. 1), suggesting that replication had extended bi-directionally to similar distances at each origin. These data indicate that the mapped *Leishmania* origins do not operate in a hierarchy of firing efficiency or timing during S phase. If only a single origin is used in each *Leishmania* chromosome, these data indicate that origin hierarchy is a coordination mechanism that arose in eukaryotes to allow multiple origins to direct replication of a single linear DNA molecule. In fact, the emergence of such non-uniform origin activity in *T. brucei* relative to *Leishmania* uniformity can be observed (Fig. 4). *T. brucei* chromosome 8, between ~0.2 and 1.5 Mb, possesses three origins of non-uniform activities; these origins are syntenically conserved with origins of uniform strength in *L. major* chromosomes 7, 10 and 23. In addition, *L. major* chromosome 31, whose chromosome copy number is >2 in all *Leishmania* species [19], is duplicated in the *T. brucei* genome on chromosomes 8 (2.0–2.5 Mb) and 4 (1.0–1.5 Mb), with origin location conserved. However, in chromosome 8 the origin is strong and colocalises with the centromere, whereas in chromosome 4 the origin is weak and non-centromeric. These data illustrate that, amongst kinetoplastid origins, even when their locations are conserved, their sequences (see below) and activity are not, reinforcing the contrast with yeast, where origin sequences (but not locations) are well conserved through evolution [4].

### Origins in *Leishmania*, but not in *T. brucei*, are found at specific genomic loci

**Fig. 5:**
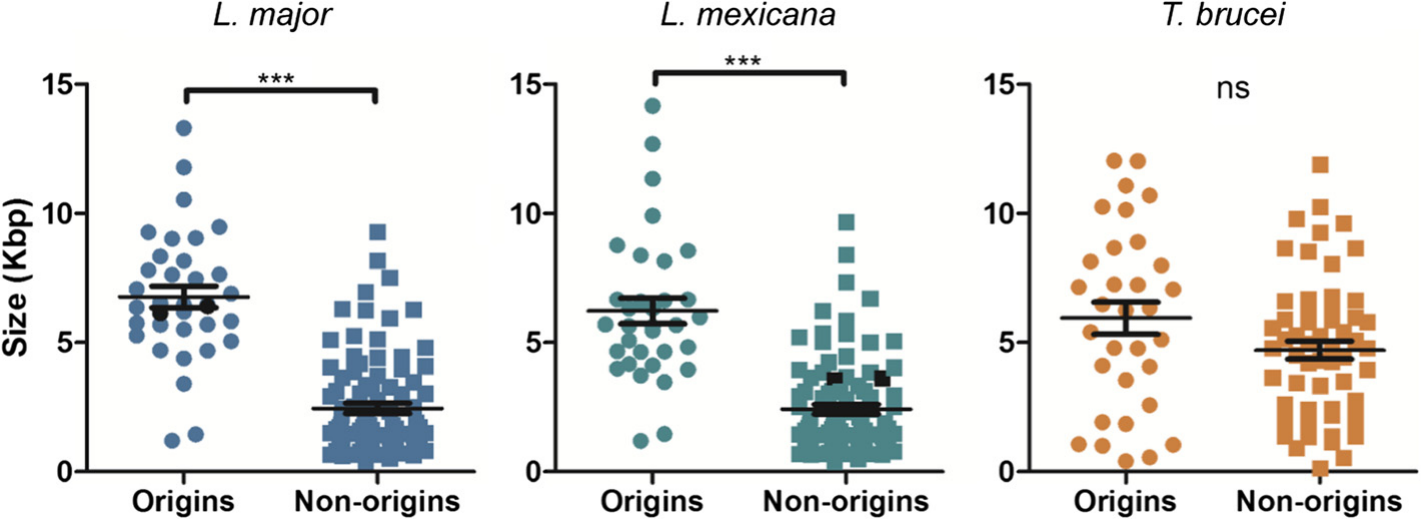
Origins are found at specific genomic loci in *Leishmania* but not in *T. brucei*. Scatter plot analysis of the length of strand switch regions (SSRs) in *L. major*, *L. mexicana* and *T. brucei*, comparing SSRs that have been mapped as showing origin activity (*circles*) with those in which origin activity has not been detected (*squares*). *Horizontal lines* show the mean, and *vertical lines* standard error of the mean; ***P < 0.0001, a significant difference in SSR size between the two groups; *ns* denotes that no significant size difference was seen. Origin-active SSRs in *L. major* chromosomes 29 and 36 are highlighted in *black*, as are the syntenic SSRs in *L. mexicana* chromosomes 8 and 20, which are not origin-active (further detail in Figure S9 in Additional file 1)

Though we have now identified the sites of DNA replication initiation in the genomes of two *Leishmania* species and in *T. brucei* [9], repeated attempts to identify common sequences (both within and between species) have to date failed (data not shown). One feature common to all origins is co-localisation with SSRs. However, origins are not found at all SSRs, and so we asked if any feature distinguishes origin-active from non-active SSRs. Figure 5 shows an analysis of SSR length in each parasite genome, measuring the distance between the start or end of the two most proximal open reading frames. In both *Leishmania* species, origin-active SSRs were significantly (*P* < 0.0001) larger than non-origin SSRs, irrespective of the configuration of transcription direction around them (Figure S8 in Additional file 1). In contrast, SSR length in *T. brucei* could not distinguish the two classes of sites. These data suggest that replication origins in *Leishmania*, but not in *T. brucei*, localise to a distinct subset of SSRs, though what features are present in the origin active sites but are absent in the other SSRs remains unclear. Focusing on the syntenic *L. mexicana* and *L. major* chromosomes that have undergone fusion or fission confirms this (Figure S9 in Additional file 1): the SSRs that display origin activity in *L. major* chromosomes 29 and 36 are notably larger than the related non-origin SSRs in *L. mexicana* chromosomes 8 and 20. These data reinforce the difference in origin function between *T. brucei* and *Leishmania* and support the suggestion that replication initiates at a single mappable origin, found within a specific SSR type, in every *Leishmania* chromosome.

## Discussion

A number of experimental strategies to map replication initiation have been applied to many eukaryotes [23], including yeast, mammals, *Arabidopsis*, *Drosophila* and *T. brucei*, and in each study multiple origins have been detected in each linear chromosome. This has led to the view that origin multiplicity is a universal feature of eukaryotic chromosome replication, distinct from single origin-based replication of the predominantly circular chromosomes in most bacteria and many archaea [1]. The work described here reveals that *Leishmania* may not conform to this view, being a eukaryote in which replication initiation is detectable at only a single, bi-directional origin in each chromosome, with each origin of equal strength and located at specific genomic loci. Below we consider the functional and evolutionary implications of these findings.

An important question posed by this work, with relevance for *Leishmania* biology, is: can each chromosome really be replicated from only one origin? *L. major* chromosomes range in size from ~0.2–2.6 Mb. In *L. mexicana* the chromosome size range is extended further, since chromosome 20 is ~3.3 Mb, larger than nine of the 11 *T. brucei* chromosomes, each of which has more than one origin [9]. It seems unlikely that the intrinsic rate of replication varies between *Leishmania* chromosomes (Fig. 1; Figures S2–S5 in Additional file 1; see below); as a result, the time to complete replication of each chromosome from one origin would vary by up to as much as 15-fold, and the time to complete genome replication would be dictated by the largest chromosome. The rate of replication fork movement has been measured to be ~3–4 kb.min^−1^ in *T. brucei* [24], which is comparable with rates of ~2–3 kb.min^−1^ in yeast [25] and other eukaryotes [2]. In *L. mexicana* S phase has been calculated as 2.9 h [26], meaning that uninterrupted progression of a bi-directional fork could replicate ~1000 kb during S phase if the lower rate estimate from *T. brucei* is applied. This prediction appears consistent with the width of the MFAseq peaks seen in late S phase *Leishmania* cells (Figures S2 and S5 in Additional file 1), though it should be noted that MFAseq is not a strategy capable of inferring replication rate with accuracy. Nonetheless, if the replication rate predictions are accurate, ~50 % of *Leishmania* chromosomes might complete replication from one origin, but the rest could not. How, then, can *Leishmania* replicate their entire genome?

MFAseq maps predominant origins in a population, and so one scenario for *Leishmania* genome duplication is that replication of each chromosome initiates mainly from a single origin, but also from multiple further origins that have escaped detection. MFAseq peak height is an indicator of the frequency with which an origin acts in the population, or the timing of activation during S phase. Modelling origin usage suggests that the MFAseq approach should be capable of detecting *Leishmania* origins that display as little as 25 % of the activity (amplitude) of the mapped origins, meaning any further origins must be activated below this threshold of detection (Figure S10 and Supplementary methods in Additional file 1). In *T. brucei* such weaker origins are readily detected by MFAseq mapping in chromosomes of comparable size to the largest *Leishmania* chromosomes (Figure S7 in Additional file 1) [9]. Thus, if further origins are present in each *Leishmania* chromosome, they must be used less frequently in the population than in *T. brucei*, despite the limited number of SSR sites at which origins localise and the pronounced synteny between the parasite genomes. If MFAseq peak height reflects the timing of origin firing during S phase, as it does in *T. brucei* (Marques et al., unpublished), it is surprising that we did not detect further peaks in late S phase cells (Figures S2 and S5 in Additional file 1). One chromosome feature that appears to replicate early in many eukaryotes is the centromere [9, 27]. Though it is currently unclear if *Leishmania* chromosomes possess discrete centromeres or where in each chromosome these features might be located (see below), it is possible that the single origin we map in each chromosome colocalises with the centromere. If so, the predominance of centromere-localised replication may mask non-centromeric origins. However, if this is correct, the focus on centromeric origins relative to the other origins must be considerable in *Leishmania*, because MFAseq in *T. brucei* readily detects both centromeric and non-centromeric replication initiation in chromosomes of comparable size (Figure S7 in Additional file 1).

To begin to ask if the mapped origins possess centromere-like features, we cloned one origin-active SSR (from chromosome 30) into the plasmid pSP72-Neo-Luc (gift, B. Papadopoulou) [28] and evaluated its stability and copy number in *L. major* relative to the ‘empty’ plasmid (Figure S11 in Additional file 1). Consistent with previous studies [29, 30], the plasmid without SSR was present in many copies (here ~90 copies) after transformation into *L. major* and could be maintained in this state by antibiotic selection. In addition, though removal of antibiotic selection led to loss of the plasmid, this was gradual over many generations (one to four plasmid copies could be detected after 200 cell divisions), consistent with some replication in the absence of an origin [29]. Addition of the origin-active SSR had two effects: plasmid copy number was substantially lower (approximately seven- to eightfold) in the antibiotic selected transformants (12–14 copies), and there was little evidence for loss of plasmid in the absence of selection (12–13 copies after 160 generations, and 7 copies after 200 generations). While these data are consistent with an origin in the chromosome 30 SSR being able to promote plasmid replication, they are not consistent with the activity of a centromere, which confers stable inheritance and single copy behaviour on plasmids in yeast [31, 32] and *Plasmodium* [33]. Nonetheless, our MFAseq origin localisation data correlate well with previous analyses that have mapped sequences needed for *Leishmania* chromosome stability. Chromosome fragmentation has been used to separate chromosomes 5 and 23 into two linear fragments, and it has been shown in each case that one fragment is stably maintained and the other lost during growth [34]; based on the origin mapping described here, in both instances the stably maintained fragment harbours the origin, whereas the unstable fragment lacks the origin (Fig. 1). In chromosome 1, related fragmentation has shown that all coding sequence, as well as the single chromosome-internal SSR, can be deleted and a stably maintained linear episome is then generated based on the ‘right’ chromosome end [35], where we have mapped the single origin (Fig. 1). In a distinct approach, directed cloning of the *Leishmania* rRNA Pol I promoter, plus some surrounding sequence, into a circular plasmid was shown to confer mitotic stably [30]. Though MFAseq cannot pinpoint origin location to the precision of a promoter element, it is again notable that the single origin for this chromosome (27 in *L. major*) maps around the rRNA promoter-containing locus (Fig. 1). Strikingly, and in contrast to what we describe here for the origin active SSR in chromosome 30 (Figure S11 in Additional file 1), addition of the RNA Pol I promoter resulted in plasmid maintenance as a single copy molecule when introduced into *Leishmania* [30]. One explanation for this discrepancy (and indeed all these data) may be that some, but not all, *Leishmania* origins colocalise with centromeres.

An alternative explanation for the apparent dichotomy between the MFAseq data and the prediction that a single origin cannot replicate all *Leishmania* chromosomes is that discrete origins beyond those we have mapped are not present, but replication of some or all *Leishmania* chromosomes is supported by initiation at non-discrete loci. In the bacterium *Escherichia coli* [36] and the archaeon *Haloferax volcanii* [37] origins can be removed from the genome and replication proceeds based on homologous recombination. In *E. coli* origin deletion is severely detrimental to growth but in *H. volcanii* it is not, and it has been argued that this is because the archaeon is polyploid [37]. The pervasiveness of genome aneuploidy in *Leishmania* may therefore be explicable mechanistically: coordination of replication initiation is based on single origins per chromosome, but as the parasite evolved larger chromosomes this alone was insufficient to expeditiously complete replication, and so recombination directs some of the reaction. A by-product of recombination-supported replication could be that supernumery chromosomes are generated periodically, and such a strategy may have been retained in evolution because it provides a means to alter gene expression and adapt to change [18]. Moreover, the use of recombination to direct some replication would be consistent with the observed genome-wide formation of episomal elements [20], and would explain why *Leishmania* supports the maintenance of virtually any extrachromosomal DNA molecule — a property that is not observed in *T. brucei* because this parasite has evolved multiple defined origins per chromosome and the machinery needed to co-ordinate their firing. Finally, if recombination-supported replication is less efficient than origin-directed replication, this may explain why S phase is around twofold longer in *Leishmania* than *T. brucei*, despite very similar genome sizes [26]. Though speculative, the suggestion that origin-independent, recombination-directed replication initiation contributes to *Leishmania* genome duplication is not without eukaryotic precedence, since autonomously replicating sequence element-independent chromosome replication has been documented in *S. cerevisiae* [38] and recombination-directed replication has very recently been suggested to contribute to the complex genome copying programmes seen during *Tetrahymena* growth [39].

If *Leishmania* chromosomes are replicated from single origins, what might this reveal about the evolution of multiple origins in eukaryotes? One possibility is that genome replication in *Leishmania* may be reflective of an ancestral eukaryote, where genome size increases initially evolved through the generation of large numbers of relatively small chromosomes, each with a single origin, rather than smaller numbers of large, multi-origin chromosomes. Several bacterial groupings have linear chromosomes that are replicated from a single *oriC*, and there is a correlation between increased genome size and linearity [40]. Furthermore, there is evidence that chromosome linearity may allow species-specific gene diversification at chromosome ends [40]. In this regard, the differing genome architecture of *T. brucei* relative to *Leishmania* may be revealing. Genome comparisons suggest that the less numerous, larger chromosomes in *T. brucei* arose by fusions of the smaller, more numerous *Leishmania* chromosomes [17]. The primary difference in gene content between the genomes is that *T. brucei* has evolved large, variable subtelomeres to house thousands of variant surface glycoprotein genes used in evasion of host adaptive immunity. Chromosome fusions might have facilitated subtelomere evolution, and the steep increase in size of each chromosome would have necessitated the use of multiple origins per molecule. If this evolutionary history is correct, it may suggest that the control circuitry needed to coordinate the single firing, per round of cell division, of multiple origins per chromosome is not present in *Leishmania*, making it a valuable model. In most eukaryotes it has proved difficult to identify origins, partly due to lack of sequence conservation, but also because replication initiator binding sites outnumber active origins, including in budding yeasts where origin sequences are highly conserved [4]. Thus, it remains only partly understood what features dictate that some potential origins are activated frequently, while others are not [14]. The difference between origin-active and inactive SSRs in *Leishmania* may provide a key tool: for instance, replication regulation factors may be recruited only to origin-active SSRs. In contrast, in *T. brucei* SSRs cannot be separated into origin active and inactive versions, suggesting that, like in other eukaryotes, all have the potential to act as origins. Comparing factors bound to origin-active and non-active SSRs in the two related parasites may reveal how the co-ordination mechanisms needed for origin multiplicity arose in eukaryotes.

## Conclusions

Mapping replication origins in *Leishmania* has revealed an unexpected divergence in origin usage relative to characterised eukaryotes, which may be unique to this genera or common amongst microbes. Understanding how *Leishmania* chromosomes are replicated will provide insight into the evolution of the machinery and co-ordination of eukaryotic DNA replication.

## Materials and methods

### Cell lines and culture, including preparation for fluorescent activated cell sorting and genomic DNA extraction

*Leishmania major* strain Friedlin and *Leishmania mexicana* strain U1103 promastigote cells were grown in modified Eagle’s medium (designated HOMEM medium, GE Healthcare) supplemented with 10 % foetal calf serum (Gibco, Life Technologies), and used for experiments at a concentration of 5 × 10^6^ cells/ml. For each cell line, approximately 1 × 10^9^ cells were collected by centrifugation for 10 min at 1000 g, washed in 1 × phosphate-buffered saline (PBS; pH 7.2) supplemented with 5 mM EDTA (Gibco, Life Technologies), and then fixed at a concentration of 2.5 × 10^7^ cells/ml (in drop-wise fashion, while gently vortexing) in 70 % methanol in 1× PBS supplemented with 5 mM EDTA. Cells were then stored at 4 °C (from overnight up to three weeks), protected from light. For each sorting session, 3 × 10^8^ fixed cells were collected by centrifugation for 10 min at 1000 g, at 4 °C, washed once in 1× PBS supplemented with 5 mM EDTA, re-suspended to a concentration of 2.5 × 10^7^ cells/ml in 1× PBS supplemented with 5 mM EDTA, 10 μg/ml of propidium iodide (Sigma Aldrich) and 10 μg/ml of RNase A (Sigma Aldrich), and incubated for 45 min at 37 °C, protected from light. The cells were then transferred through a 35 μm nylon mesh cell strainer cap to a BD Falcon™ tube (BD Biosciences), and sorted into G1, early S, late S and G2 phases by fluorescent activated cell sorting (FACS) using a FACSAria I™ cell sorter (BD Biosciences). Sorted cells were collected at 4 °C into lysis buffer (1 M NaCl, 10 mM EDTA, 50 mM Tris–HCl pH 8.0, 0.5 % SDS, 0.4 mg/ml proteinase K, and 0.8 μg/ml of glycogen; Azuara 2006), incubated for 2 h at 55 °C, and stored at −20 °C. Genomic DNA (gDNA) was extracted using a Blood and Tissue DNA extraction kit (Qiagen), by omitting the lysis steps of the manufacturer’s protocol. For both sequencing and quantitative PCR real-time (qPCR), gDNA concentrations were measured using Qubit® 2.0 Fluorometer (Life Technologies).

### DNA library preparation, sequencing and marker frequency analysis

The DNA libraries were prepared using the Nextera® XT DNA Sample Preparation kit (Illumina), and subsequently sequenced using Illumina MiSeq paired-end 250-bp sequencing system (Illumina). The samples were multiplexed, with each of the early S, late S, G1 and G2 samples per species/strain sequenced in the same run to eliminate differences due to batch effects. The resulting data were analysed for quality control using FastQC [41], then trimmed using fastq-mcf (ea-utils [42]) to exclude the adapter sequences. The reads were next aligned to the respective reference genomes (TriTrypDB version 6.0) using Bowtie2 (version 2.2.0 --very-sensitive-local -k1) [43]. The aligned reads were then compared using essentially the method described previously [9], but simplified to facilitate inter-species comparisons: reads were binned in 2.5-kb sections along each chromosome, and the number of reads in each bin then used to calculate the ratios between early S versus G2/G1 and late S versus G2 samples, scaled for the total size of the read library (reads per 2.5 kb per million reads mapped). These data were then represented in a graphical form using ggplot2 and the R package (version 3.0.2 [44]). Shell scripts used to generate these data are available from [45].

### Marker frequency analysis by qPCR

A strategy employed previously [9] was used, and the assays were planned according to MIQE guidelines [46]. Primers were designed for several regions across *L. major* chromosomes 8, 20, 29 and 36, as well as *L. mexicana* chromosomes 8 and 20, using Primer Express version 3.0 (BioRad), and according to suggested guidelines [47] for primers to be used in qPCR. Primer sizes ranged from 17–24 bp, with melting temperatures from 58–60 °C, resulting in amplicons of 55–113 bp with melting temperatures from 79–85 °C. Primer efficiency and specificity were assessed for all pairs of primers by the analysis of calibration curves and melting profiles, respectively, which resulted in efficiencies of approximately 100 %, all within a 10 % interval. For normalization, the *L. major* gene LmjF.36.1980 (equivalent to LmxM.36.1980 in *L. mexicana*) was chosen as the reference gene, since the MFAseq data suggested it is in a non-origin region that is not yet replicated in early S phase. For each pair of primers, triplicates of each sample (early S, late S and G2 phases) were run per plate (MicroAmp® Optical 96-well Reaction Plate, Life Technologies), which were sealed with MicroAmp® clear adhesive film (Life Technologies). SYBR Select Master Mix (Life Technologies) was used, together with 400 nM of primers (Eurofins MWG Operon, Ebersberg, Germany) and 0.01 ng of sample gDNA, to a total of 20 μl per reaction. All experiments were run in a 7500 Real Time PCR system (Applied Biosystems), using the following PCR cycling conditions: 50 °C for 2 min and 95 °C for 2 min, followed by 40 cycles of 95 °C for 15 s, 59 °C for 15 s, and 72 °C for 1 min. Fluorescence intensity data were collected at the end of the extension step (72 °C for 1 min), after which a final dissociation step was included in order to confirm the specificity of the reaction. The resulting fluorescence intensity data were then analysed by relative quantification using the ΔΔC_t_ method [48] (7500 software version 2.3, Applied Biosystems), with the G2 phase sample being used as the calibrator. Graphs were generated using GraphPad Prism version 5.03. Primers (Table 1) targeting regions of the following genes (gene ID as presented in TritrypDB [49]) in *L. major* (LmjF) and *L. mexicana* (LmxM) were used: LmjF.29.0810, LmjF.29.0930, LmjF.29.0030, LmjF.29.2060, LmjF.08.0090, LmjF.08.1000, LmjF.08.0260, LmjF.08.0360, LmjF.36.1900, LmjF.36.3790, LmjF.36.2830, LmjF.36.3000, LmjF.20.0705, LmjF.20.1210, LmjF.20.1530; LmxM.08_29.0810, LmxM.08_29.0930, LmxM.08_29.0030, LmxM.08_29.2060, LmxM.08.0090, LmxM.08.1000, LmxM.08.0260, LmxM.08.0360, LmxM.36.1900, LmxM.36.3790, LmxM.36.2830, LmxM.36.3000, LmxM.20.0705, LmxM.20.1210, and LmxM.20.1530.

**Table 1:** Sequences of the primers used for marker frequency analysis-qPCR

Gene ID	Sense	Sequence
LmjF.29.0030/LmxM.08_29.0030	Fwd	CGATGTCGGGACTTACGTAAAGT
	Rev	TCCACAGCGTGTATCCTTTCG
LmjF.08.0090/LmxM.08.0090	Fwd	CAGCCTCTACCGCGTCTTTC
	Rev	TCTCCTTCAGTCGGACGTATGTC
LmjF.29.0810/LmxM.08_29.0810	Fwd	CATCATGATCAAGACCCTCGAGTA
	Rev	GGCGACTTCGCAGCTTCTC
LmjF.29.0930/LmxM.08_29.0930	Fwd	ACTCGACTGCGCCTCATTG
	Rev	TGACAGGAGAGGGACGAAGAG
LmjF.29.2060/LmxM.08_29.2060	Fwd	AGCCACCTTTAACGCCATTGT
	Rev	GGAACAGGAGGCCATCGAA
LmjF.08.0260/LmxM.08.0260	Fwd	CAACAAGTCGGCCACTTACAAG
	Rev	CGCCACATCTGCCATGAG
LmjF.08.0360/LmxM.08.0360	Fwd	CCCTCCGCCACAATGAG
	Rev	TTCGCCCACGCTAGTATCG
LmjF.08.1000/LmxM.08.1000	Fwd	GGAACCTGACCTACCCCTTCTC
	Rev	GTCGAAGTTGAAGACGTTGTTGA
LmjF.36.1900/LmxM.36.1900	Fwd	CCACACACTCGCCTCTTACTACA
	Rev	AGCTCAGGGTCACGCAAAAG
LmjF.36.2830/LmxM.36.2830	Fwd	TGCGGAGCGCAAGAATG
	Rev	GGCGAGGCGGAACATCT
LmjF.36.3000/LmxM.36.3000	Fwd	TGTGGGAGGAAACAATCAGCTT
	Rev	GTGGCGGAGAGGAAAACGTA
LmjF.36.3790/LmxM.36.3790	Fwd	GCACACACGGTACTGCTTCAA
	Rev	CACGGGCTAAGCGCACTAG
LmjF.20.0705/LmxM.20.0705	Fwd	TGGGCTAGCTCCTTCTTTCACT
	Rev	TTCGTCCTTGAGCTTGTACTTGAC
LmjF.20.1210/LmxM.20.1210	Fwd	GTCGCCGCAACCAGTACAT
	Rev	CCGGAGAAGTGCTGGTACA
LmjF.20.1530/LmxM.20.1530	Fwd	TCCGCTGTTTGACGTGTATAGC
	Rev	TCAACTCCTCCACCTTGCATATC
LmjF.36.1980/LmxM.36.1980	Fwd	GAGGTTCATGAGCTTGGGTTTAA
	Rev	TGCAAGGGAACAGGTGGTTT

Both *L. major* and *L. mexicana* gene IDs are shown. *Fwd* denotes forward strand, *Rev* denotes reverse strand

### SSR size analysis

SSRs containing origins were identified, and viewed on ‘genome browser’ using the TriTrypDB version 8.0 [49] database platform. The distance between the two most proximal genes to the SSR (divergent, convergent or head-to-tail) was measured by subtracting the coordinates of the stop or start codon of the gene to the left of the SSR from the coordinates of the stop or start codon of gene on the right. The same was performed for other SSRs, where origins were not identified. The size of the distance between genes at the SSRs was then plotted onto a vertical scatter plot using GraphPad Prism version 5.03. Statistical significance was inferred by employing a non-parametric, single-tailed, Mann–Whitney test, with a *p* value threshold of <0.05.

### Data access

MFAseq data are being hosted at TriTryDB [49] and are currently scheduled for release in Autumn 2015. Sequence data have been deposited in the European Nucleotide Archive [50], accession number [ENA:PRJEB7849]).

## Additional file

Additional file 1:
**Supplementary methods and supporting information (11 figures and 1 table, each of which is referred to and explained in the main paper).** (PDF 68032 kb)

## Abbreviations

bp: base pair
gDNA: genomic DNA
H-T: head-to-tail
PBS: phosphate-buffered saline
qPCR: quantitative PCR
RNA Pol: RNA polymerase
SSR: strand switch region
