# Supplementary material for: Genome-wide mapping reveals single-origin chromosome replication in Leishmania, a eukaryotic microbe
Source: Genome Biol. 2015 Oct 19;16:230. doi: 10.1186/s13059-015-0788-9 (PMC4612428; doi:10.1186/s13059-015-0788-9)
Supplement: Additional file 1: — Supplementary methods and supporting information (11 figures and 1 table, each of which is referred to and explained in the main paper). (PDF 68032 kb) [file 13059_2015_788_MOESM1_ESM.pdf]

Genome-wide mapping reveals single-origin chromosome replication in *Leishmania*, a eukaryotic microbe

Catarina A. Marques, Nicholas J. Dickens, Daniel Paape and Richard McCulloch

Supplementary methods

Modelling Origin Usage

In order to simulate the distribution of reads if non-mapped origins were present at potential locations of origins (strand-switch regions; SSRs) we used *L. major* chromosome 36, since this has the most potential origins, being the largest chromosome in this species. We used a bespoke python script to read the distribution of reads at the natural origin (position LmjF.36: 1110127-1116528) and add reads with this distribution to all the SSR locations in the chromosome (positions LmjF.36: 155190-156279, LmjF.36:778681-779754, LmjF.36:1413340-1414163, LmjF.36:1607311-1608348, LmjF.36:2078727-2082697, LmjF.36:2468381-2470493). The distribution of inserted reads at each position was added to the normal (non-origin) distribution of the reads to try and mitigate any position read depth effects. The bam files that were generated were then merged using samtools to create a bam file with a single origin used in 100%, or with 6 additional, non-natural origins each with 17% usage. This merged bam file was then processed using the MFAseq pipeline and it was clear that these origins would not have been easily visible in our analysis (data not shown).

To derive a threshold of visible detection of origins we took a file with a single simulated origin (position LmjF.36: 155190-156279) and merged it multiple times with the original *L. major* early-S data to dilute the percentage of usage. Each of this series of 'dilutions' (no dilution, 5:1, 2:1, 1:1, 1:2 and 1:5, plus the original early-S track) was plotted using the MFAseq pipeline and these results are show in Figure S10. Using the natural, mapped origin of smallest amplitude (*L. major* chromosome 4) we then measured the height of each peak using a bespoke script to measure the median ratio for the SSR and the median ratio for the whole chromosome (median so as not to exaggerate heights). The resulting ratio was 1.037 (origin/chromosome), so we set this is a stringent limit of reasonable detection. We then plotted the median for the simulated and diluted origin, showing there was a good trend and fit to the data (Fig.S10), and so we used this to

calculate the percentage 'dilution' at which we would have a ratio of 1.037. The region background is 1.038 and the threshold of detection is consequently 1.076, which corresponds to a dilution of 25.09%. We therefore declared the stringent threshold of visible detection as 25%. The scripts used in this analysis are freely and publicly available from: http://bitbucket.org/WTCMPCPG/originssimulation/.

Plasmid generation and transformation

The origin-active strand switch region (SSR) on chromosome 30 from *L. major* Friedlin was amplified from genomic DNA (primers Fw: GAC*ATTAAT*CCGCTGATACCAGGTGTGAG; Rev AC*ATTAAT*AGACACTGAAGTTCCACGGCAG; AseI restriction sites italicized and underlined) and cloned into the NdeI site of pSP72-Neo-Luc plasmid [1]. *L. major* Friedlin promastigote cells (5×10^{7} cells) were transformed by nucleofection as described by Castanys-Muñoz and colleagues [2] with 10 µg pSP72-Neo-Luc-origin or pSP72-Neo-Luc plasmid DNA. Transformant clones were selected in medium containing 25 $\mu g.ml^{-1}$ G418.

in vitro luciferase assay

L. major Friedlin transformants were grown in the absence or presence of 25 $\mu g.ml^{-1}$ G418 and at indicated generation times 5×10^{6} cells were pelleted and washed twice with PBS. Cells were then resuspended in 100 µl PBS and transferred into a well of a white 96 well microplate (Gibco). After addition of 100 µl D-luciferin (150 $\mu g.ml^{-1}$, reconstituted in Mg/Ca-free Dulbecco's modified PBS) (Promega) light emission was measured (PHERAstar FS, BMG Labtech) for 2h, with measurements taken every 5 min. Values plotted were from the 90 min time point.

Quantitative PCR analysis

L. major Friedlin transformants were grown in the absence or presence of 25 $\mu g.ml^{-1}$ G418 and at the indicated generations 2×10^{7} cells were pelleted and DNA was extracted with DNeasy Blood and Tissue Kit (Qiagen). DNA concentration was then determined by Quant-iT PicoGreen dsDNA assay (LifeTechnologies), according to manufactures instructions. qPCR was performed in a 25 µl total volume with 3 ng of DNA, Power Sybr Green (Applied Biosystems). The following primers were used for the quantification of the G418 resistance gene on the pSP72 plasmids: Fw, TGCCTGCTTGCCGAATATCA; Rev, ATATCACGGGTAGCCAACGC). As a reference, a control region on chromosome 36 was PCR-amplified (see LmjF.36.1980; primer pair in Table 1). qPCR was

performed as described in the main text. To determine the plasmid copy number we normalized to the chromosome reference (ΔCT = CTG418 - genomic CTgDNA; plasmid copy number = 2 × 2(-ΔCT)).

Supplementary figure legends

Fig. S1. Fluorescence Activated Cell Sorting of *Leishmania major* cells. The diagram shows a representative fluorescence profile of propidium iodide-stained cells and the locations (double-headed arrows) of the gates used to sort G1 (diploid, 2N), early-S, late-S and G2 (4N) phase cells.

Fig.S2. Mapping late replication origins in the *L. major* nuclear genome. Graphs show the distribution of replication origins in the 36 chromosomes of *L. major* (numbered 1-36; sizes denoted in intervals of 0.25 Mb), determined by the extent of enrichment of DNA in late S phase cells relative to G2. For each chromosome, the top track displays CDSs, with genes transcribed and translated from right to left in red, and from left to right in blue. The graph below shows the ratio of the read-depth between the late S phase and G2 samples (y-axis), where each dot (light blue) represents the median S/G2 ratio (y-axis) in a 2500 bp window across the chromosome (x-axis). Finally, the track below the graph displays localization of acetylated histone H3 (H3ac) in each chromosome (data from [3]), identifying positions of transcription start sites (y-axis: values represented as log2 in 250 bp windows).

Fig.S3. Comparing S/G1 and G2/G1 read depth ratios in the *L. major* nuclear genome. The 36 *L. major* chromosomes (numbered 1-36) are depicted as in Fig.S2 and Fig.1, with CDS organisation in the top panel, enrichment of H3Ac along the chromosomes in the bottom panel and the size of each chromosome indicated in intervals of 0.25 Mb (x-axes). For each chromosome, the upper graph (purple) shows Early S/G1 DNA sequence depth ratios (y-axis), while the lower graph shows the G2/G1 DNA sequence depth ratios (grey); each dot represents the median ratio in a 2500 bp window across the chromosome.

Fig.S4. Comparing early replication origins in the *L. mexicana* and *L. major* nuclear genomes. Graphs show the distribution of replication origins in the 34 chromosomes of *L. mexicana* (numbered 1-34; sizes denoted in intervals of 0.25 Mb), relative to the 36 *L . major* chromosomes. Highlighted in red are *L. mexicana* chromosomes 8 and 20, each of which is syntenic with two *L.*

major chromosomes (29 and 8, and 36 and 20, respectively). Early S/G2 DNA sequence depth ratios (*L. major* blue, *L. mexicana* green) and CDS organisation are as detailed in Fig.1 and Fig.S2. Acetylated histone H3 (H3ac) localisation is shown for *L. major* (as in Fig.1 and Fig.S2), but has not been mapped in *L. mexicana*.

Fig.S5. Comparing early and late replication origins in the *L. mexicana* nuclear genomes. Graphs show the distribution of replication origins in the 34 chromosomes *L. mexicana* (numbered 1-34; sizes denoted in intervals of 0.25 Mb). Early S/G2 DNA sequence depth ratios (green) are shown relative to late S/G2 ratios (yellow) and to CDS organisation, as detailed in Fig.1 and Fig.S2.

Fig.S6. Genome-wide origin conservation between *L. major* and *T. brucei*, using the *T. brucei* chromosomes as framework. The 11 chromosomes of *T. brucei* are shown, within which CDS organisation (genes transcribed and translated from right to left in red, and from left to right in blue) and localisation of histone H4, acetylated on lysine reside 10, are indicated (black tracks, based on [4]). Regions of synteny between the 11 *T. brucei* and the 36 *L. major* chromosomes are indicated by showing the *L. major* chromosome numbers below. Note, that some *L. major* chromosomes are completely syntenic with parts of the *T. brucei* molecules, whereas others are fragmented; in addition, for simplicity, we have not detailed the relative orientation of the synteny blocks: readers are referred to [5] for greater detail. Conservation of replication origins between the two genomes is detailed as follows: blue denotes origins in loci that are conserved in the two genomes; red denotes loci that are conserved between the genomes but only show origin activity in *T. brucei*; dotted pink denotes loci that are conserved between the genomes but only show origin activity in *L. major*; burgundy denotes a *T. brucei* origin at the site of chromosome rearrangement between the genomes, with no detectable origin activity in *L. major*; grey denotes a *T. brucei* origin in a region of unclear synteny due to local rearrangements relative to the *L. major* chromosome; black denotes an origin within a subtelomeric region of *T. brucei* chromosome 6 that is not found in the *L. major* genome; boxes denote origins that are coincident with mapped centromeres (chromosomes 1-8).

Fig.S7. Comparing MFAseq profiles in *L. major* and *T. brucei*. Graphs show the distribution of replication origins in chromosome 8 of *T. brucei* (Tb) and in the three largest chromosomes of *L. major* (Lmj34, 35 and 36). In all cases chromosome sizes (x-axes) are denoted in intervals of 0.25 Mb, and early S/G2 DNA sequence depth ratios (*L. major* blue, *T. brucei* orange; y-axes) and CDS

organisation are as detailed in Fig.1 and Fig.S2. The approximate location of the mapped centromere (green circle) in *T. brucei* chromosome 8 is indicated.

Fig.S8. Strand switch size and origin activity in *Leishmania*. Scatter plot analysis of the length of strand switch regions (SSRs) in *L. major* (blue) and *L. mexicana* (green), comparing SSRs that have been mapped as showing origin activity (circles) with those in which origin activity has not been detected (squares). Here, the SSRs have been separated into distinct configurations of the surrounding genes: DSS denotes where the directional gene clusters (DGCs) to the left and right of the SSR are each transcribed away from the SSR; h-t denotes that one DGC is transcribed towards the SSR and the other away; and CSS denotes that both surrounding DGCs are transcribed towards the SSR. Horizontal lines show the median, and vertical lines standard deviation.

Fig.S9. Detailed analysis of two syntenic strand switch regions displaying differing origin activity between *L. mexicana* and *L. major*. Two diagrammatic representations are shown, comparing (upper) *L. mexicana* (Lmx) chromosome 20 with *L. major* chromosome 36, and (lower) *L. mexicana* chromosome 8 and *L. major* chromosome 29. In each case, blocks of synteny are boxed and their relative orientation indicated, and early-mid S/G2 DNA sequence read depth ratios (*L. mexicana* green, *L. major* blue) and CDS organisation are shown as in Fig.2. Dotted boxes identify strand switch regions (SSRs) that are conserved, but show origin activity in *L. major* and not in *L. mexicana*. In each case, further details of the SSRs are shown below, representing the two most proximal genes (arrows denote expression direction) on either side, with the relative sizes of the SSRs indicated by dotted lines. In the whole chromosomes sizes are denoted by intervals of 0.25 Mb, whereas the detailed SSR diagrams show sizes in Kb (numbered from the start of the leftmost gene).

Fig.S10. MFAseq of *L. major* chromosome 36 with a second, simulated origin data. The relative visibility of a simulated (S) origin (at location ~0.2 Mb) in *L. major* chromosome 36 is shown relative to the natural (N) origin (~1.1 Mb), comparing MFAseq peaks in which the simulated origin is used at different percentages of the population (determined by percentage of reads that have that particular distribution in the early S sample). The simulated 'origin' is visible at 33% but not distinguishable (by eye) from background at 17%. CDS organisation (top track), chIP enrichment of H3Ac (bottom track) and the MFAseq tracks (comparing real and simulated read depth in S/G2) are shown as in Fig.S2. The inset shows a graph of the S/G2 peak medians for the simulated origin

on *L. major* chromosome 36 at the different percentages; the horizontal line shown at 0.953 is the chromosome, non-origin S/G2 median for *L. major* chromosome 36 in the simulation.

Fig.S11. Analysis of bacterial plasmid stability in *Leishmania major*. (A) Depiction of plasmid pSP72-Neo-Luc [1], in which the origin active strand switch region (SSR) from chromosome 30 was cloned and its maintenance in *L. major* compared with the same plasmid without added SSR. Only relevant plasmid features are shown: the G418 resistance gene ($G418^R$) used to select for transformed *L. major* promastigotes; the luciferase gene used to assess plasmid retention; and the ampicillin resistance gene and ColE1 origin used for plasmid propagation in *E. coli.* **(B)** Luciferase levels (relative light units) were determined for two clones of *L. major* transformed with the SSR-containing plasmid (+SSR) or the plasmid without the SSR (-SSR); values are shown after increasing generations of growth in the absence of G418 selection and are expressed as a percentage of the values for the same clones grown (for the same number of generations) on G418 selection. **(C)** Analysis of copy number (determined by qPCR of the $G418^R$ gene relative to a locus on *L. major* chromosome 36) of the SSR-containing plasmid (+SSR) or the plasmid alone (-SSR), in each case comparing two *L. major* transformant clones. Values are shown for plasmid copy number after establishing the transformant clones by G418 selection (0 generations) and after 160 or 200 generations of subsequent growth in the presence (+ drug selection) or absence (- drug selection) of G418.

Table S1. Minimal and maximal median S/G2 ratios, derived from 2.5 kbp bins after plotting sequence reads from DNA derived from early S and G2 phase cells, for the 36 *L. major* chromosomes; data are shown either for the whole chromosome, for the reads spanning the origin-active SSR, or for a 30 kbp region around the origin-defining MFAseq peak.

* in chromosome 1 (LmjF.01) there is no clearly defined origin-active SSR, and so the SSR region is arbitrarily defined as the region between the two genes proximal to the right-hand telomere (see Fig.1)

References

1. Roy G, Dumas C, Sereno D, Wu Y, Singh AK, Tremblay MJ, Ouellette M, Olivier M, Papadopoulou B: **Episomal and stable expression of the luciferase reporter gene for**

quantifying Leishmania spp. infections in macrophages and in animal models. *Mol Biochem Parasitol* 2000, **110:**195-206.

2. Castanys-Munoz E, Brown E, Coombs GH, Mottram JC: **Leishmania mexicana metacaspase is a negative regulator of amastigote proliferation in mammalian cells.** *Cell Death Dis* 2012, **3:**e385.
3. Thomas S, Green A, Sturm NR, Campbell DA, Myler PJ: **Histone acetylations mark origins of polycistronic transcription in Leishmania major.** *BMC Genomics* 2009, **10:**152.
4. Siegel TN, Hekstra DR, Kemp LE, Figueiredo LM, Lowell JE, Fenyo D, Wang X, Dewell S, Cross GA: **Four histone variants mark the boundaries of polycistronic transcription units in Trypanosoma brucei.** *Genes Dev* 2009, **23:**1063-1076.
5. El Sayed NM, Myler PJ, Blandin G, Berriman M, Crabtree J, Aggarwal G, Caler E, Renauld H, Worthey EA, Hertz-Fowler C, et al: **Comparative genomics of trypanosomatid parasitic protozoa.** *Science* 2005, **309:**404-409.

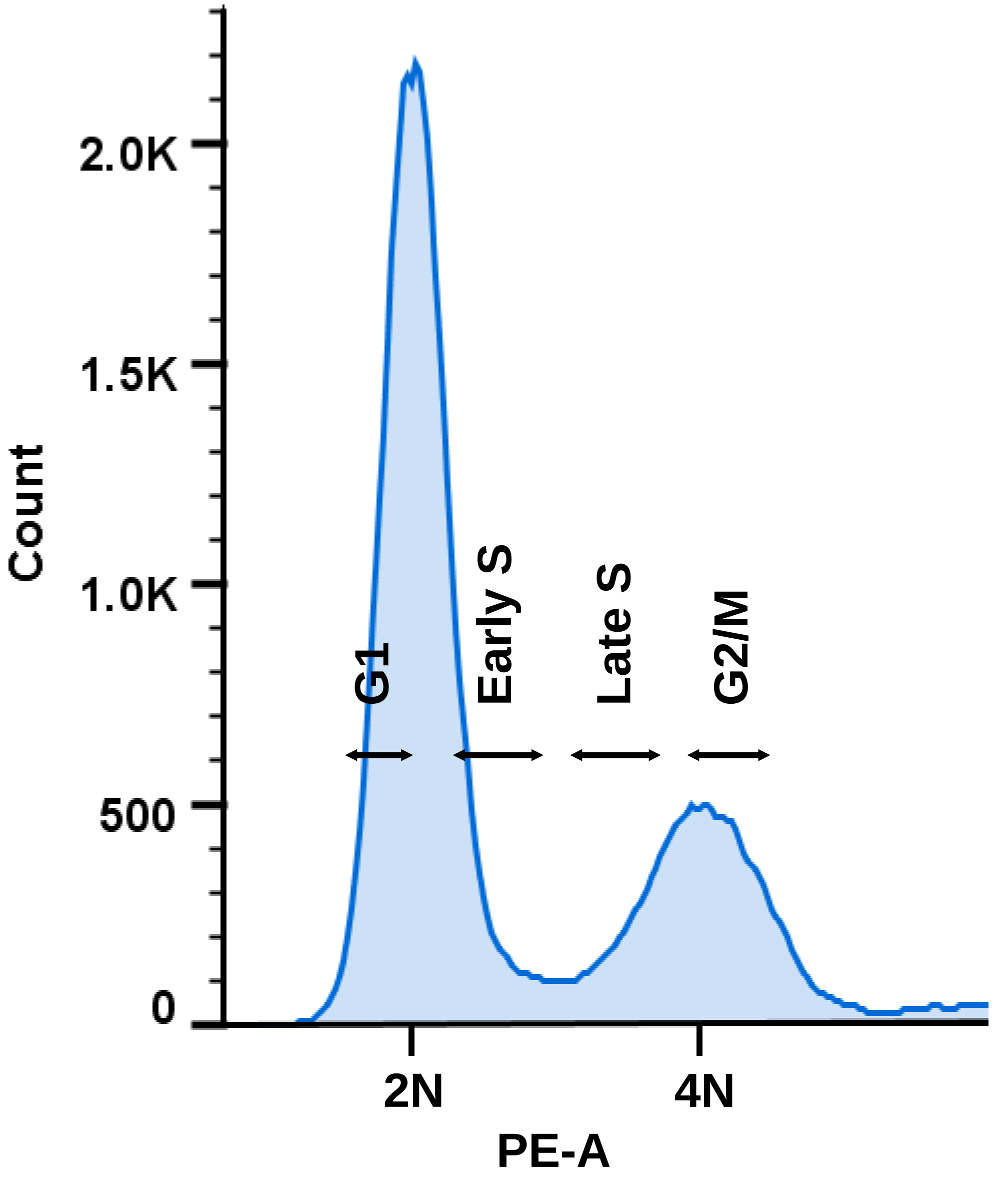

Fig.S1

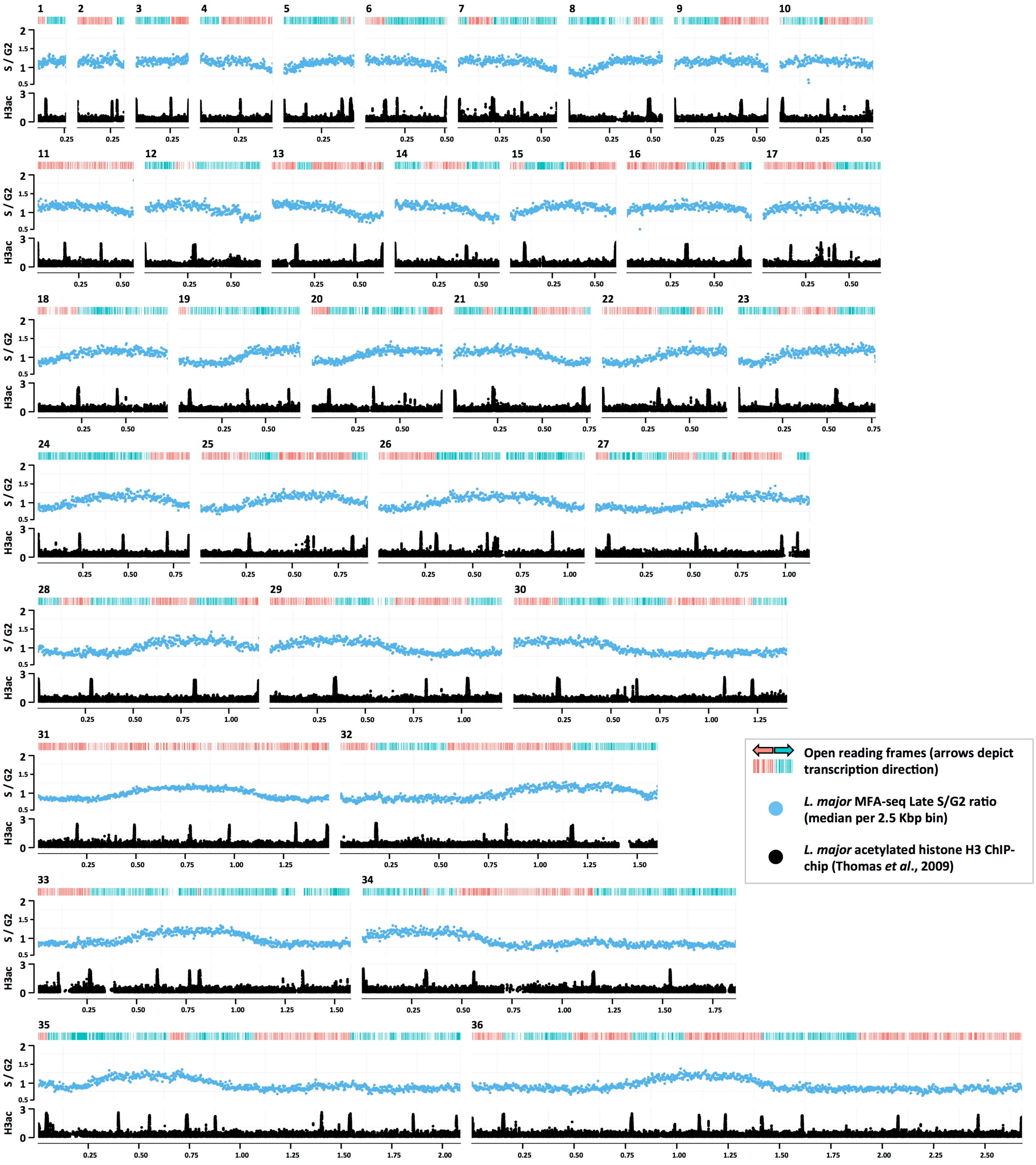
S / G2
H3ac
Open reading frames (arrows depict transcription direction)
L. major MFA-seq Late S/G2 ratio (median per 2.5 Kbp bin)
L. major acetylated histone H3 ChIP-chip (Thomas *et al.*, 2009)

Fig.S2

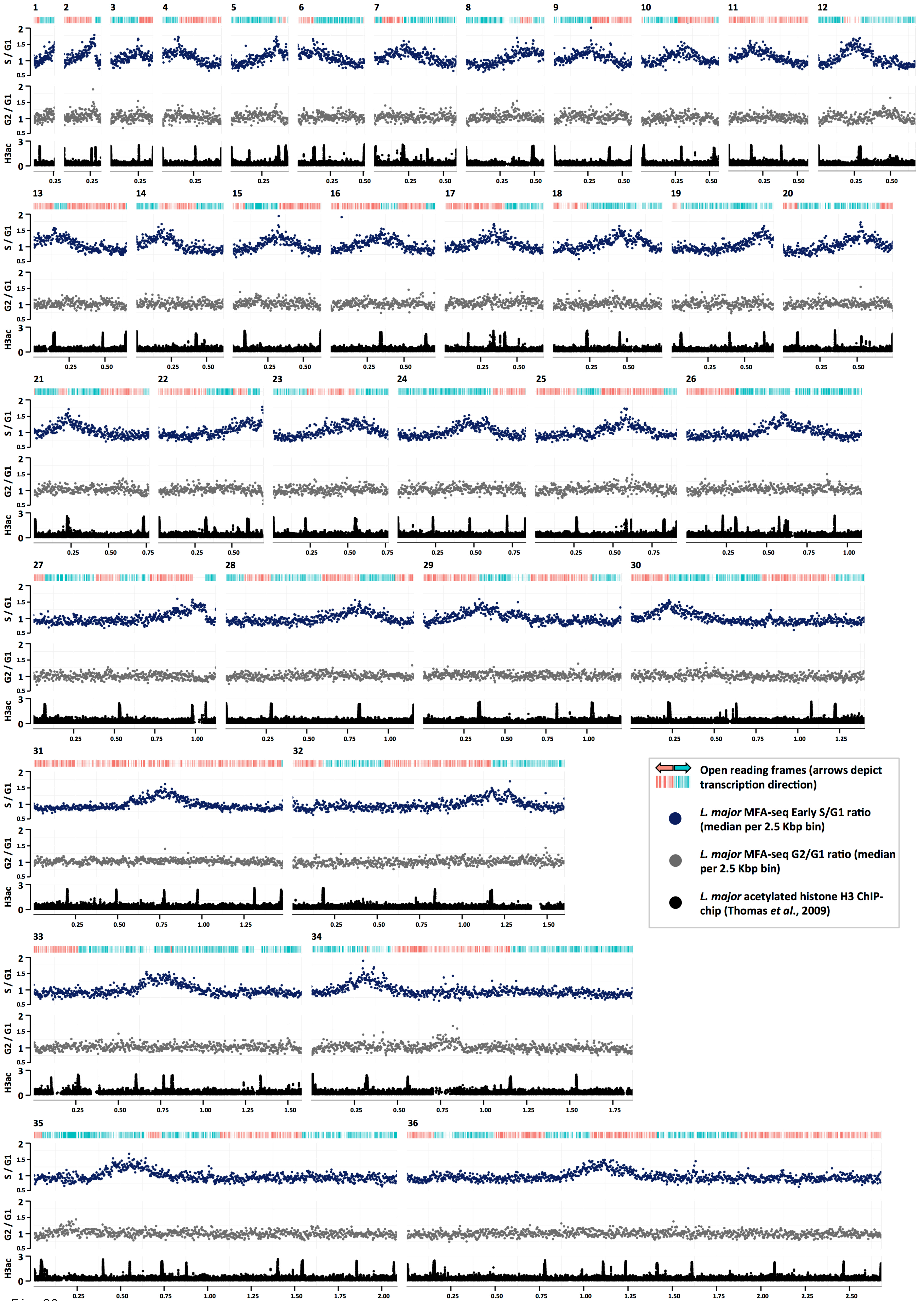
S / G1
G2 / G1
H3ac
Open reading frames (arrows depict transcription direction)
L. major MFA-seq Early S/G1 ratio (median per 2.5 Kbp bin)
L. major MFA-seq G2/G1 ratio (median per 2.5 Kbp bin)
L. major acetylated histone H3 ChIP-chip (Thomas et al., 2009)

Fig.S3

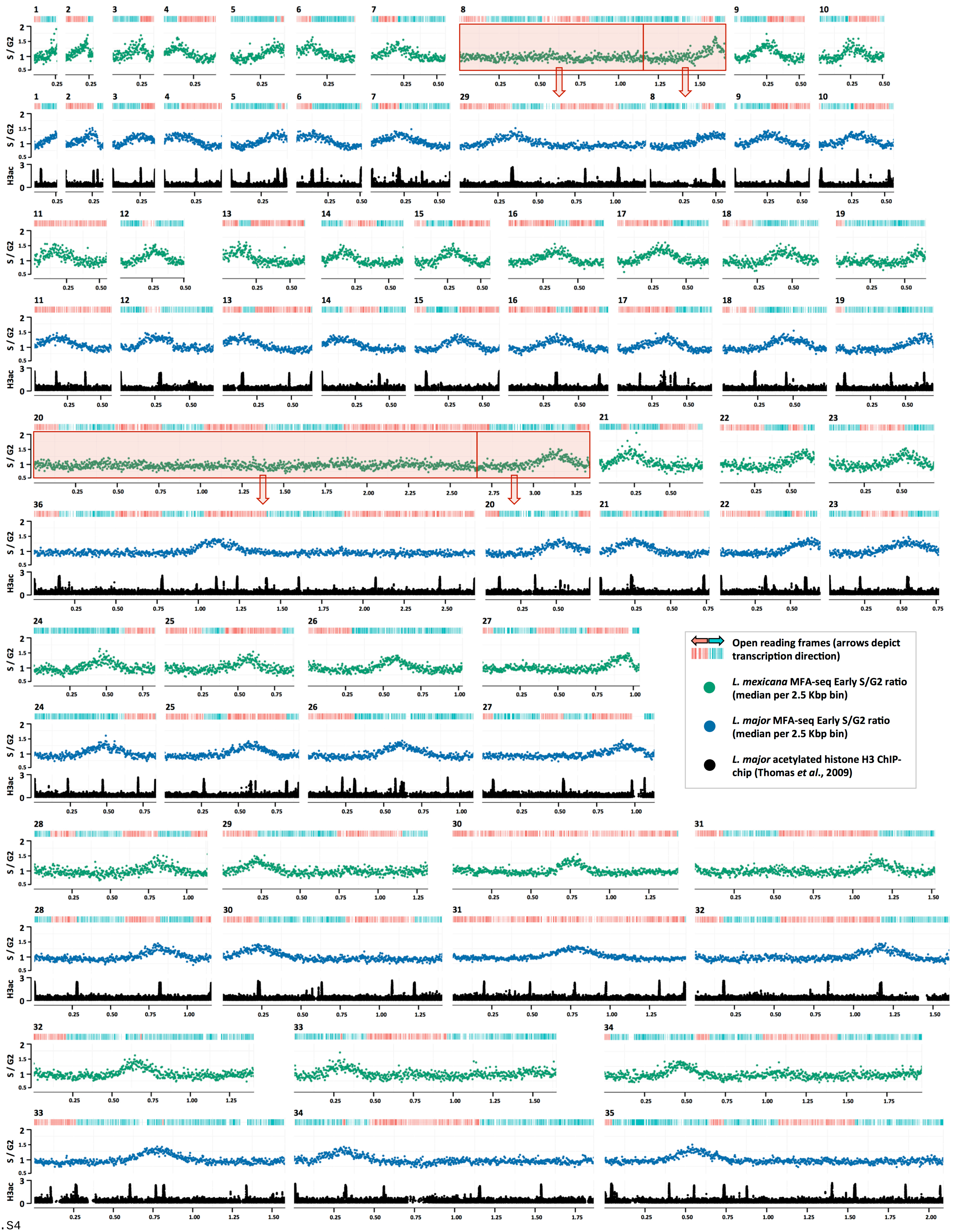
Open reading frames (arrows depict transcription direction)
L. mexicana MFA-seq Early S/G2 ratio (median per 2.5 Kbp bin)
L. major MFA-seq Early S/G2 ratio (median per 2.5 Kbp bin)
L. major acetylated histone H3 ChIP-chip (Thomas et al., 2009)
S / G2
H3ac

Fig.S4

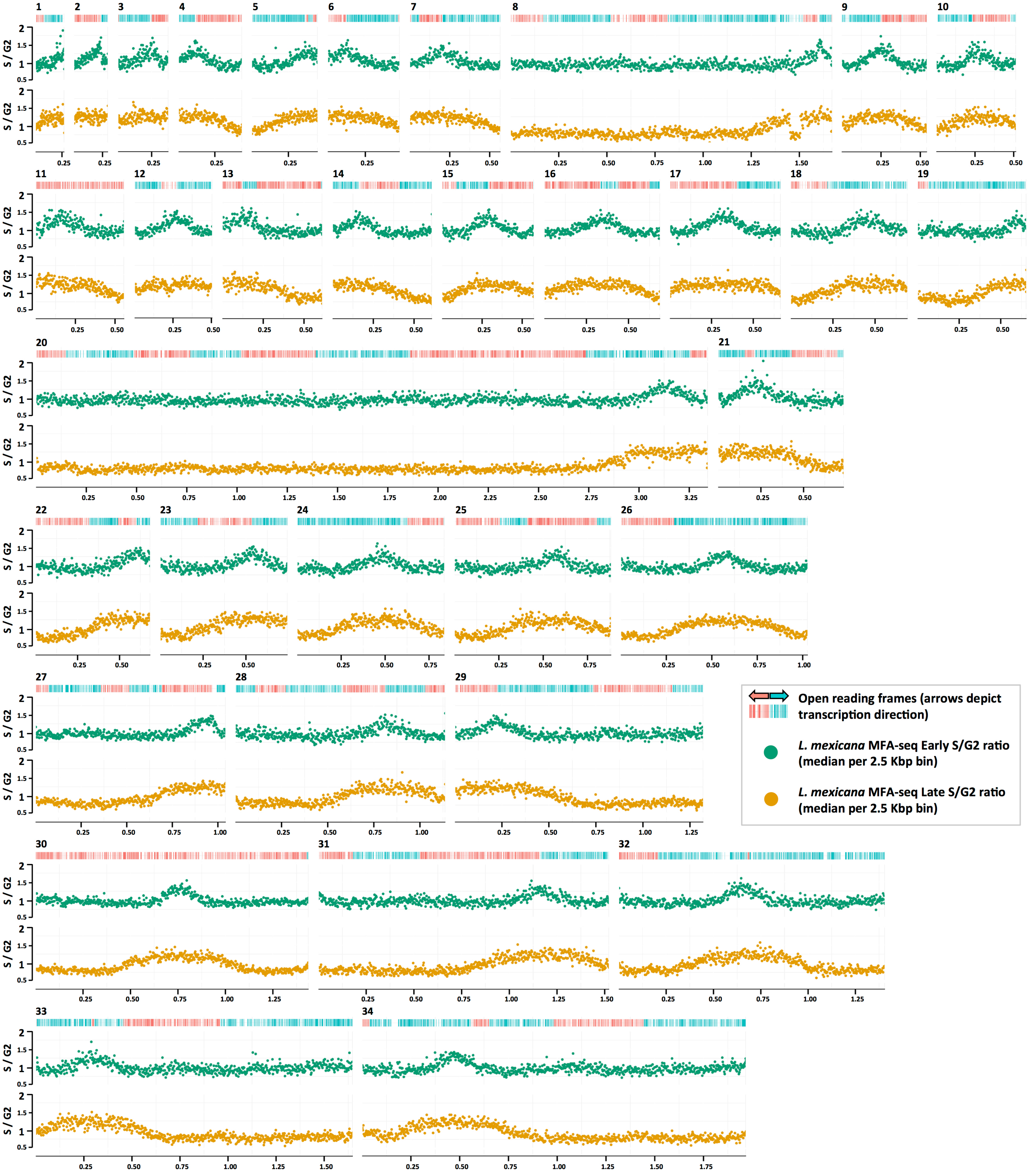
Open reading frames (arrows depict transcription direction)
L. mexicana MFA-seq Early S/G2 ratio (median per 2.5 Kbp bin)
L. mexicana MFA-seq Late S/G2 ratio (median per 2.5 Kbp bin)
S / G2

Fig.S5

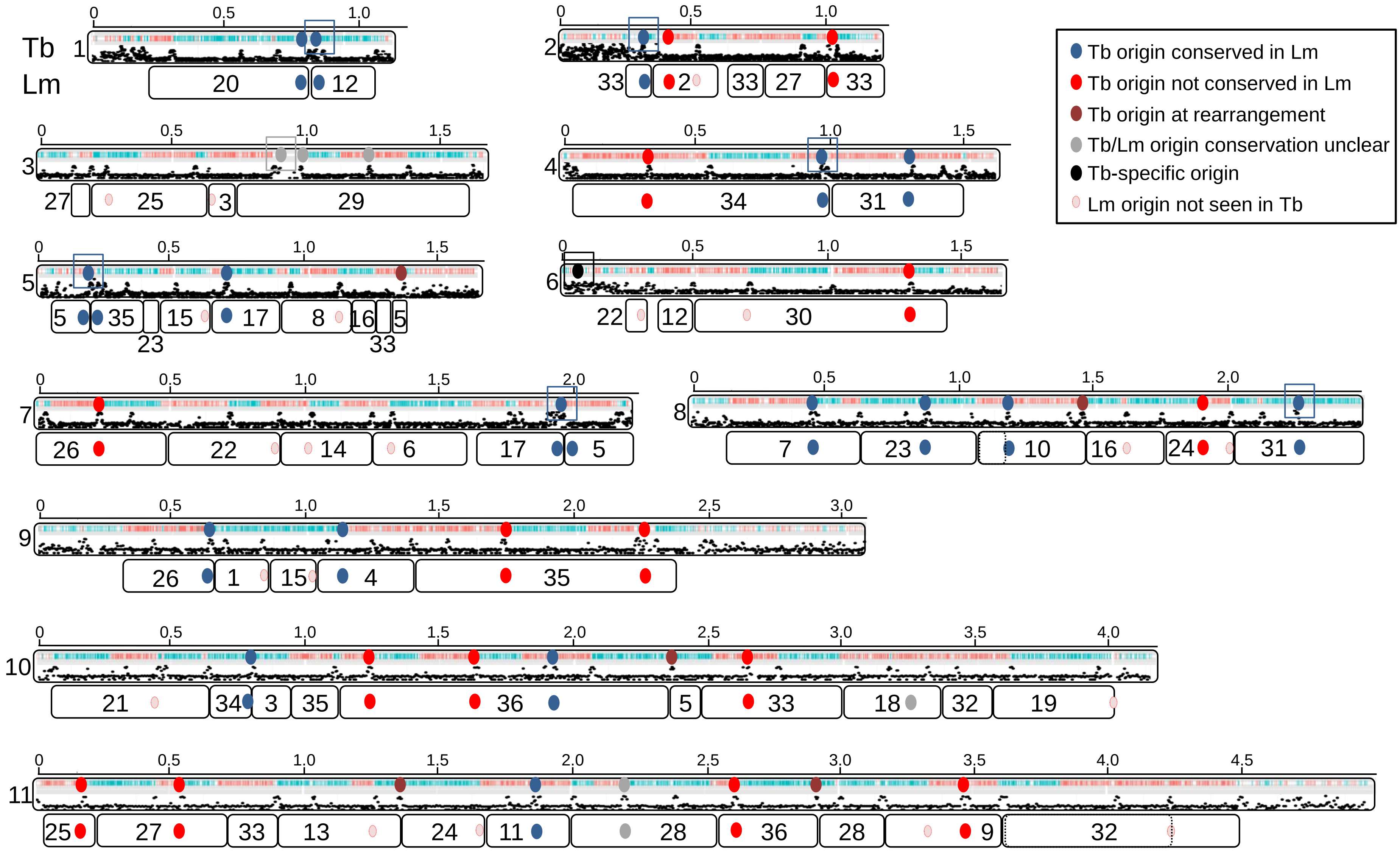
Tb
Lm
Tb origin conserved in Lm
Tb origin not conserved in Lm
Tb origin at rearrangement
Tb/Lm origin conservation unclear
Tb-specific origin
Lm origin not seen in Tb

Fig.S6

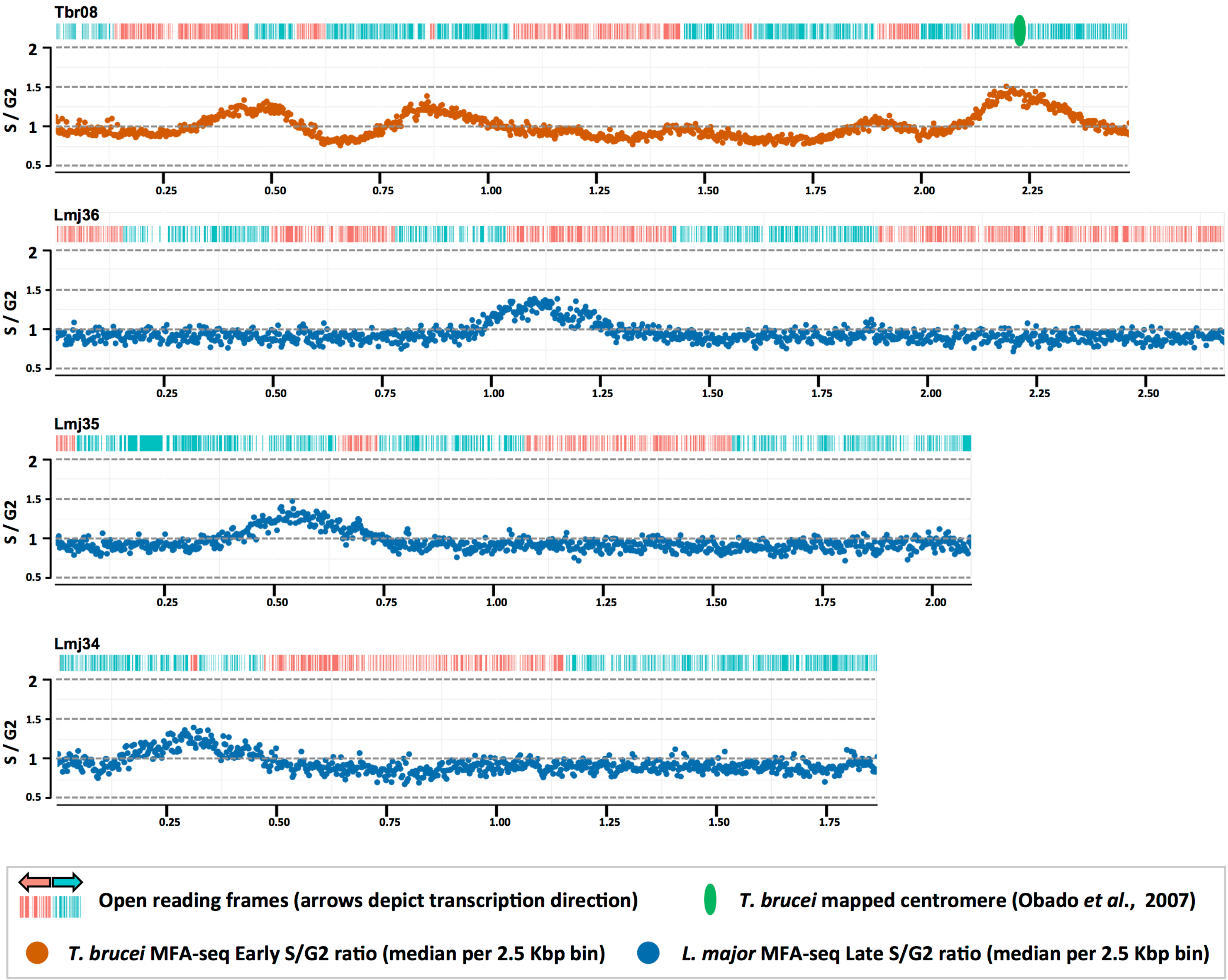
Tbr08
S / G2
2
1.5
1
0.5
0.25
0.50
0.75
1.00
1.25
1.50
1.75
2.00
2.25
Lmj36
2.50
Lmj35
Lmj34
Open reading frames (arrows depict transcription direction)
T. brucei mapped centromere (Obado et al., 2007)
T. brucei MFA-seq Early S/G2 ratio (median per 2.5 Kbp bin)
L. major MFA-seq Late S/G2 ratio (median per 2.5 Kbp bin)

Fig.S7

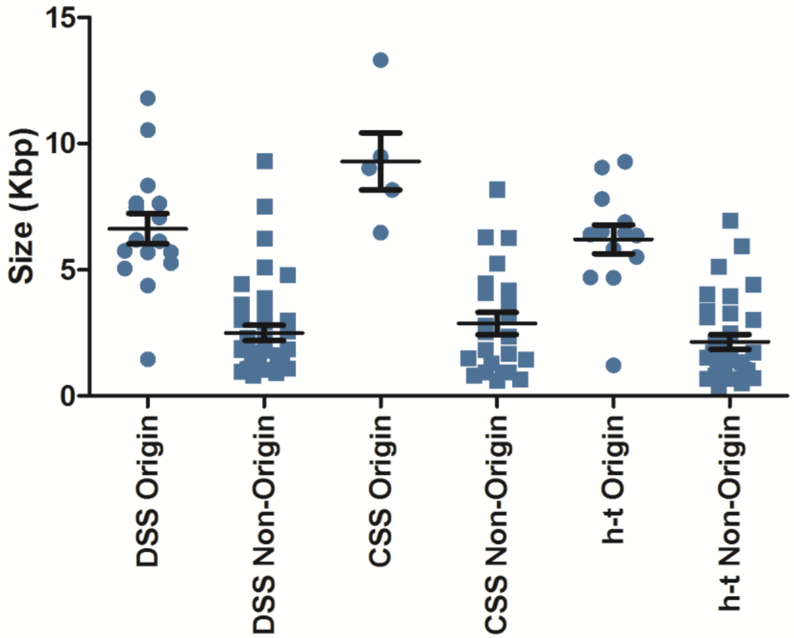

15
10
5
0
Size (Kbp)
DSS Origin
DSS Non-Origin
CSS Origin
CSS Non-Origin
h-t Origin
h-t Non-Origin

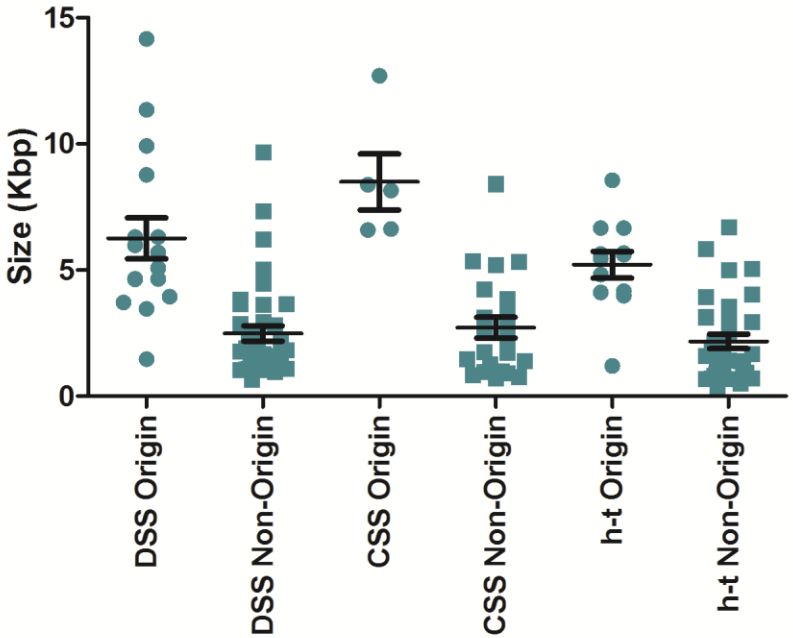

15
10
5
0
Size (Kbp)
DSS Origin
DSS Non-Origin
CSS Origin
CSS Non-Origin
h-t Origin
h-t Non-Origin

Fig.S8

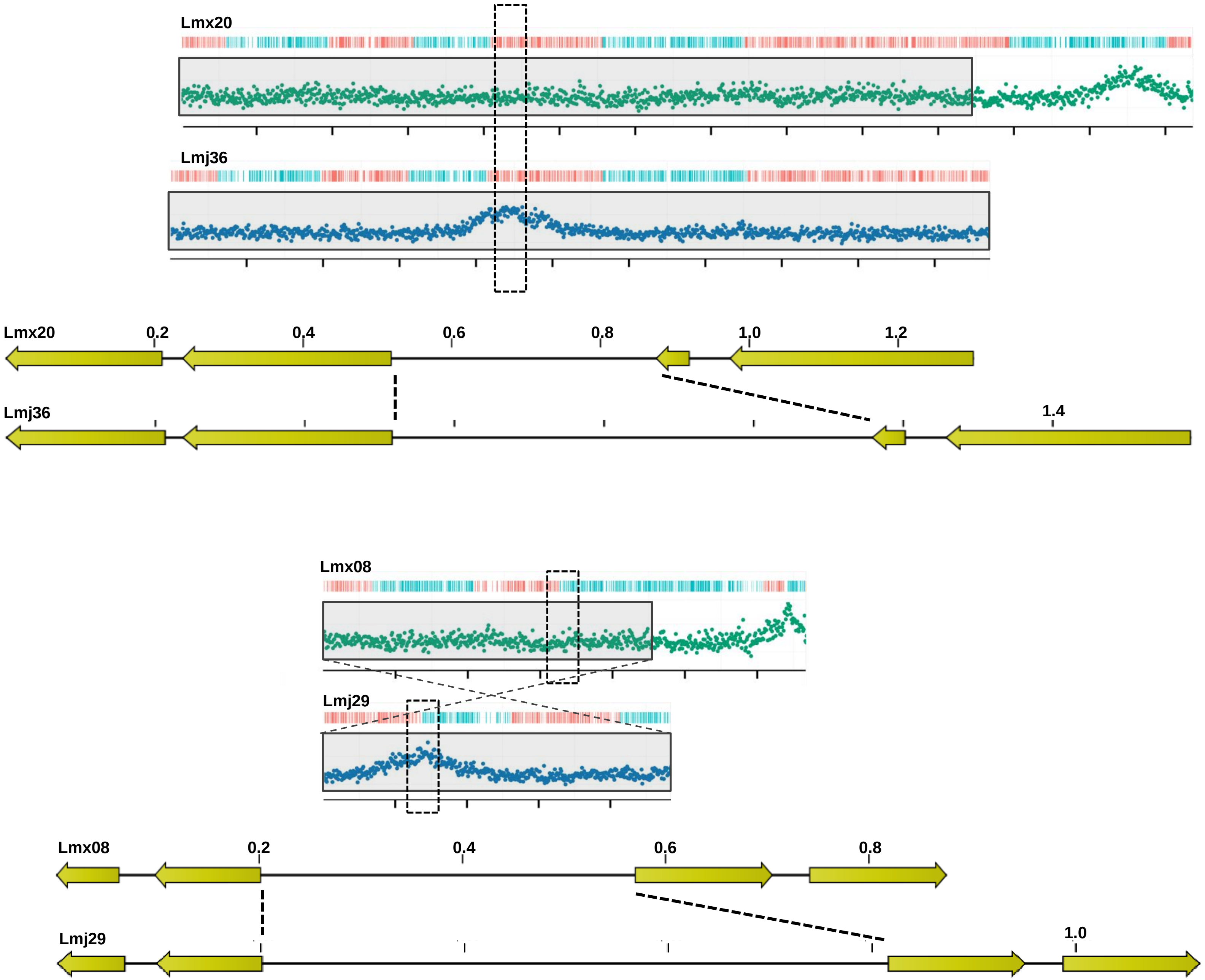

Lmx20
Lmj36
Lmx20
0.2
0.4
0.6
0.8
1.0
1.2
Lmj36
1.4
Lmx08
Lmj29
Lmx08
0.2
0.4
0.6
0.8
Lmj29
1.0

Fig.S9

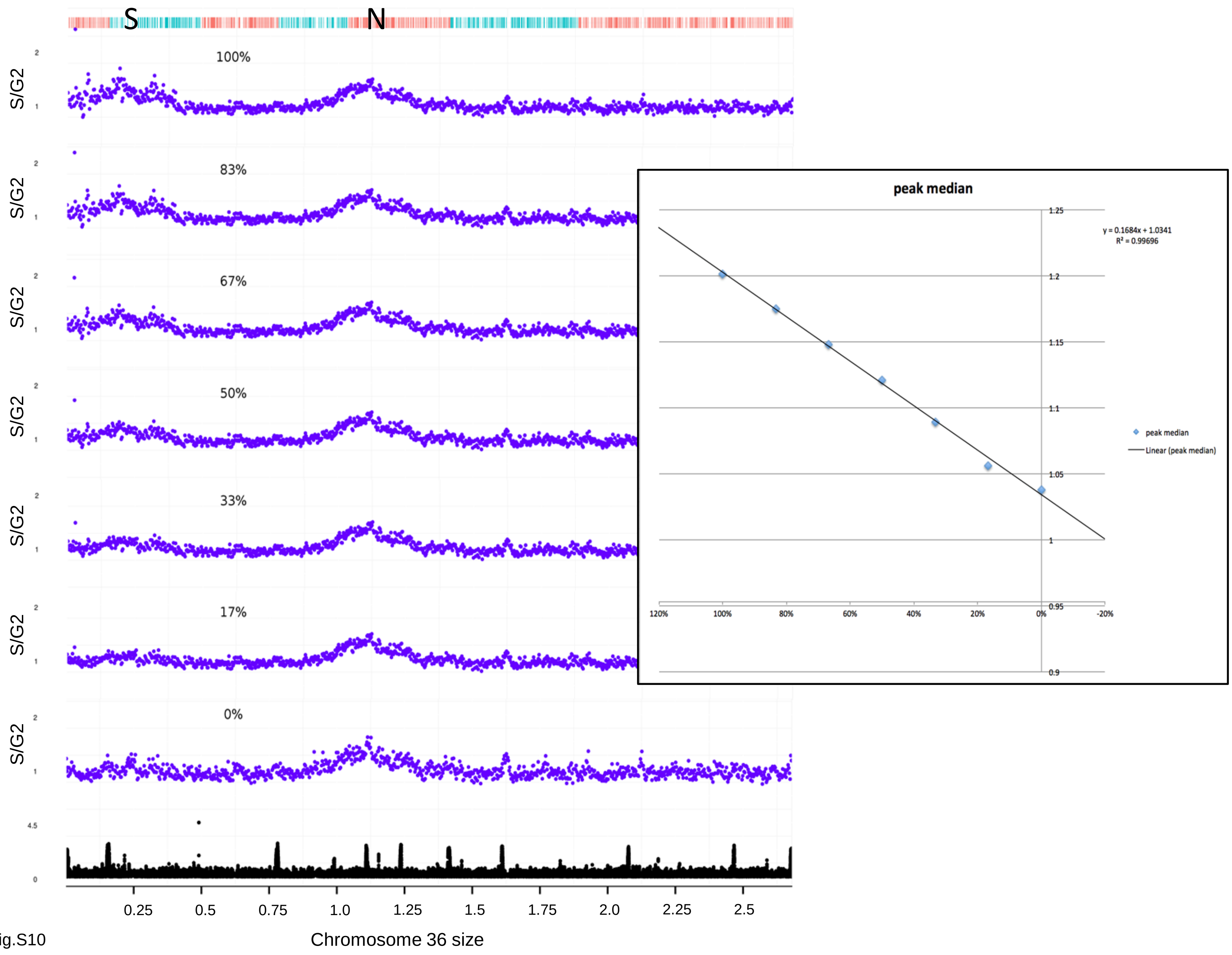
S
N
100%
83%
67%
50%
33%
17%
0%
S/G2
2
1
4.5
0
0.25
0.5
0.75
1.0
1.25
1.5
1.75
2.0
2.25
2.5
Chromosome 36 size
peak median
y = 0.1684x + 1.0341
R² = 0.99696
1.25
1.2
1.15
1.1
1.05
1
0.95
0.9
120%
100%
80%
60%
40%
20%
0%
-20%
peak median
Linear (peak median)

Fig.S10

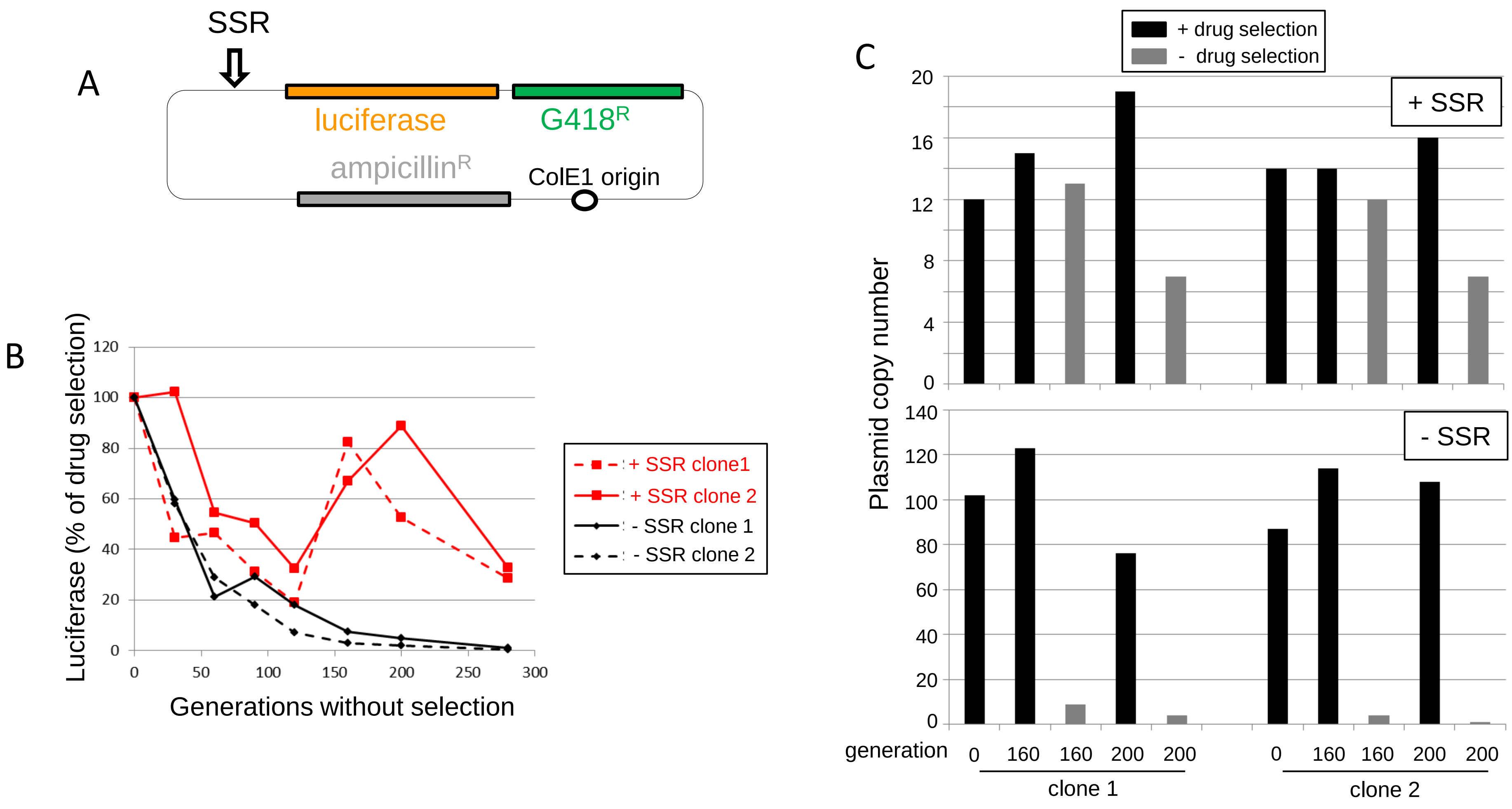
A
SSR
luciferase
G418R
ampicillinR
ColE1 origin
B
Luciferase (% of drug selection)
Generations without selection
+ SSR clone1
+ SSR clone 2
- SSR clone 1
- SSR clone 2
C
+ drug selection
- drug selection
Plasmid copy number
+ SSR
- SSR
generation
0 160 160 200 200
0 160 160 200 200
clone 1
clone 2

Fig.S11

earlyS vs G2	*Whole Chromosome*			*Origin SSR*			*Origin Region (30kb)*		
**Chromosome**	**Min**	**Max**	**Median**	**Min**	**Max**	**Median**	**Min**	**Max**	**Median**
LmjF.01	0.839	1.353	1.041	1.222*	1.251*	1.2365*	1.11*	1.353*	1.243*
LmjF.02	0.844	1.436	1.042	1.205	1.436	1.376	1.064	1.436	1.308
LmjF.03	0.815	1.306	1.075	1.042	1.306	1.227	1.021	1.306	1.193
LmjF.04	0.781	1.335	1.067	1.059	1.269	1.161	1.059	1.333	1.208
LmjF.05	0.757	1.471	1.0045	1.331	1.471	1.401	1.117	1.471	1.277
LmjF.06	0.774	1.471	1.0525	1.329	1.458	1.4045	1.17	1.471	1.357
LmjF.07	0.756	1.42	1.057	1.128	1.337	1.306	1.128	1.42	1.307
LmjF.08	0.715	1.458	0.963	1.152	1.458	1.38	1.152	1.458	1.346
LmjF.09	0.739	1.398	1.036	1.252	1.381	1.3195	1.09	1.381	1.254
LmjF.10	0.797	1.97	1.042	1.174	1.268	1.254	1.174	1.379	1.258
LmjF.11	0.794	1.456	1.078	1.15	1.456	1.36	1.15	1.456	1.315
LmjF.12	0.689	1.633	0.9595	1.235	1.481	1.3455	1.235	1.481	1.358
LmjF.13	0.748	1.563	0.997	1.291	1.494	1.475	1.257	1.563	1.346
LmjF.14	0.794	1.41	1.008	1.127	1.332	1.179	1.127	1.345	1.234
LmjF.15	0.747	1.436	0.976	1.105	1.294	1.21	1.105	1.336	1.272
LmjF.16	0.746	1.529	1.007	1.342	1.529	1.424	1.204	1.529	1.323
LmjF.17	0.77	1.499	1.015	1.253	1.481	1.338	1.116	1.499	1.261
LmjF.18	0.712	1.437	1.032	1.123	1.391	1.322	1.123	1.434	1.336
LmjF.19	0.744	1.539	0.9645	1.166	1.371	1.309	1.156	1.539	1.354
LmjF.20	0.76	1.558	0.998	1.159	1.558	1.447	1.13	1.558	1.403
LmjF.21	0.775	1.493	0.999	1.228	1.453	1.375	1.195	1.493	1.355
LmjF.22	0.738	1.692	0.9625	1.271	1.321	1.279	1.258	1.471	1.321
LmjF.23	0.75	1.502	1.036	1.21	1.469	1.272	1.176	1.502	1.272
LmjF.24	0.748	1.365	0.959	1.25	1.327	1.306	1.135	1.363	1.243
LmjF.25	0.757	1.455	0.957	1.283	1.366	1.3245	1.151	1.455	1.291
LmjF.26	0.754	1.508	0.9315	1.322	1.352	1.33	1.314	1.508	1.372
LmjF.27	0.775	1.48	0.955	1.248	1.395	1.328	1.214	1.423	1.337
LmjF.28	0.765	1.473	0.9535	1.385	1.403	1.394	1.231	1.473	1.353
LmjF.29	0.753	1.465	0.952	1.138	1.408	1.364	1.138	1.465	1.321
LmjF.30	0.761	1.507	0.943	1.376	1.495	1.483	1.288	1.507	1.417
LmjF.31	0.77	1.387	0.942	1.163	1.359	1.313	1.163	1.359	1.296
LmjF.32	0.741	1.559	0.944	1.252	1.442	1.372	1.252	1.559	1.315
LmjF.33	0.726	1.454	0.926	1.237	1.454	1.315	1.237	1.454	1.305
LmjF.34	0.64	1.46	0.938	1.312	1.459	1.438	1.173	1.46	1.312
LmjF.35	0.705	1.49	0.9445	1.288	1.49	1.451	1.202	1.49	1.329
LmjF.36	0.769	1.458	0.9405	1.329	1.434	1.413	1.255	1.458	1.376
Column mean	0.76	1.48	0.99	1.23	1.41	1.34	1.17	1.45	1.31
Column median	0.76	1.47	0.99	1.24	1.43	1.34	1.16	1.46	1.32

Table S1
